# The use of acupuncture for addressing neurological and neuropsychiatric symptoms in patients with long COVID: a systematic review and meta-analysis

**DOI:** 10.3389/fneur.2024.1406475

**Published:** 2024-07-19

**Authors:** Wai Ching Lam, Dongjue Wei, Huijuan Li, Liang Yao, Shiping Zhang, Michael X. Y. Lai, Ya Zheng, Jerry W. F. Yeung, Alexander Y. L. Lau, Aiping Lyu, Zhaoxiang Bian, Angela M. Cheung, Linda L. D. Zhong

**Affiliations:** ^1^Biomedical Sciences and Chinese Medicine, School of Biological Sciences, Nanyang Technological University, Singapore, Singapore; ^2^Chinese Medicine Teaching and Research Division, School of Chinese Medicine, Hong Kong Baptist University, Hong Kong SAR, China; ^3^Department of Health Research Methods, Evidence, and Impact, McMaster University, Hamilton, ON, Canada; ^4^Kwong Wah Hospital - The Chinese University of Hong Kong Chinese Medicine Clinical Research and Services Centre, Tung Wah Group of Hospitals, Hong Kong SAR, China; ^5^Faculty of Health and Social Sciences, School of Nursing, The Hong Kong Polytechnic University, Hong Kong SAR, China; ^6^Division of Neurology, Department of Medicine and Therapeutics, Faculty of Medicine, The Chinese University of Hong Kong, Hong Kong SAR, China; ^7^Faculty of Medicine, Gerald Choa Neuroscience Centre, Lui Che Woo Institute of Innovative Medicine, The Chinese University of Hong Kong, Hong Kong SAR, China; ^8^Hong Kong Chinese Medicine Clinical Study Centre, School of Chinese Medicine, Hong Kong Baptist University, Hong Kong SAR, China; ^9^Department of Medicine, University Health Network, University of Toronto, Toronto, ON, Canada

**Keywords:** acupuncture, long COVID, fatigue, depression, anxiety, headache, insomnia, cognitive impairment

## Abstract

**Importance:**

Acupuncture has been used to treat neurological and neuropsychiatric symptoms in China and other parts of the world. These symptoms, such as fatigue, headache, cognitive impairment, anxiety, depression, and insomnia, are common in people experiencing long COVID.

**Objective:**

This study aims to explore the feasibility of acupuncture in the treatment of neurological and neuropsychiatric symptoms in long COVID patients.

**Data Sources:**

A systematic search was conducted in four English and four Chinese databases from inception to 23 June 2023. Literature selection and data extraction were conducted by two pairs of independent reviewers.

**Study Selection:**

Randomized controlled trials (RCTs) that explored the effect of acupuncture on fatigue, depression, anxiety, cognitive abnormalities, headache, and insomnia were included.

**Data Extraction and Synthesis:**

RCTs that explored the effect of acupuncture on fatigue, depression, anxiety, cognitive abnormalities, headache, and insomnia were included. A meta-analysis was performed using R software. Heterogeneity was measured using I^2^. Subgroup analyses were performed focusing on the duration of treatment and acupuncture modalities. The systematic review protocol was registered on PROSPERO (registration number: CRD42022354940).

**Main outcomes and measures:**

Widely adopted clinical outcome scales included the Fatigue Scale for assessing fatigue, the Hamilton Depression Rating Scale for evaluating depression, the Mini-Mental State Examination for assessing cognitive impairment, the Visual Analog Scale for headache severity, and the Pittsburgh Sleep Quality Index for measuring insomnia.

**Results:**

A total of 110 RCTs were included in the systematic review and meta–analysis. Overall, acupuncture was found to improve the scores of the Fatigue Scale (vs. medication: mean differences (MD): −2.27, *P* < 0.01; vs. sham acupuncture: MD: −3.36, *P* < 0.01), the Hamilton Depression Rating Scale (vs. medication: MD: −1.62, 95%, *P* < 0.01; vs. sham acupuncture: MD: −9.47, *P* < 0.01), the Mini–Mental State Examination (vs. medication: MD: 1.15, *P* < 0.01; vs. sham acupuncture: MD: 1.20, *P* < 0.01), the Visual Analog Scale (vs. medication: MD: −1.05, *P* < 0.01; vs. waitlist: MD: −0.48, P=0.04), and the Pittsburgh Sleep Quality Index (vs. medication: MD: −2.33, *P* < 0.01; vs. sham acupuncture: MD: −4.19, *P* < 0.01).

**Conclusion and relevance:**

This systematic review suggested acupuncture as a potentially beneficial approach for the treatment of neurological and neuropsychiatric symptoms, as assessed using clinical scales, and it may have applicability in long COVID patients. Further well-designed clinical studies specifically targeting long COVID patients are needed to validate the role of acupuncture in alleviating long COVID symptoms.

**Systematic Review Registration:**

PROSPERO, identifier [CRD42022354940].

## Key points

- **Question:** Would acupuncture be potentially beneficial for neurological and neuropsychiatric symptoms in long COVID patients?
- **Findings:** In this meta-analysis of 110 studies, it was found that acupuncture, compared to medication or sham acupuncture, could improve the measurement scores of symptoms, including fatigue, depression, cognitive impairment, headache, and insomnia, among long COVID patients. Approximately 7.8% of patients who received acupuncture treatment reported the incidence of adverse effects.
- **Meaning:** This systematic review suggested that acupuncture could be explored as an alternative approach for addressing neurological and neuropsychiatric complications in long COVID patients.

## 1 Introduction

Approximately 10–30% of people have lingering symptoms after an acute SARS-CoV-2 infection, making it challenging for them to return to work and resume their normal daily activities (1). These include neurological symptoms such as fatigue, cognitive impairment, brain fog, and loss of smell and neuropsychiatric symptoms such as anxiety and depression, as well as physical symptoms such as dyspnea, cough, myalgia, and arthralgia—a phenomenon colloquially termed “long COVID” (2–6).

One of the most significant impairments in patients with long COVID are neurological and neuropsychiatric symptoms (7). In a meta-analysis of more than 10,000 patients selected from 18 published studies, nearly one-third of patients experienced neurological and neuropsychiatric symptoms such as fatigue, cognitive dysfunction, and sleep disturbances, 3 months after the onset of acute COVID-19 infection (8). Similarly, another review that studied neuropsychiatric symptoms of COVID-19 found depression, anxiety, cognitive abnormalities, fatigue, sleep disturbances, and headache to be the most commonly reported long COVID-related neuropsychiatric symptoms reported between 4 weeks and 6 months after the onset of acute COVID-19 infection (9).

Given the unclear pathophysiology of long COVID, physicians have been using clinical strategies adapted from the treatment of similar symptoms/conditions with some measure of success (10). Acupuncture, a common technique for treating neurological and neuropsychiatric symptoms in traditional Chinese medicine, has been widely used in clinical practice. There is an increasing body of evidence showing that acupuncture may help alleviate neurological and neuropsychiatric symptoms not only in patients with acute pain (11) but also in those with specific chronic conditions (12–15), as well as those with severe diseases such as cancer-related insomnia and fatigue (16, 17).

Although previous systematic reviews have suggested acupuncture as an effective and safe treatment for patients with various neurological and neuropsychiatric disorders, these studies are not appropriate for providing evidence for long COVID as they included patients with multiple primary diseases or chronic conditions. Contrarily, many of the long COVID patients were generally well before the onset of acute COVID infection. To provide insights into the management of a newly emerging condition, our systematic review aimed to summarize and analyze the efficacy and safety of acupuncture in treating the common neurological and neuropsychiatric symptoms that are found in long COVID patients, namely fatigue, insomnia, depression, anxiety, cognitive abnormalities, and headache, based on data from randomized controlled trials (RCTs).

## 2 Methods

### 2.1 Literature search

We performed the systematic review in accordance with the Preferred Reporting Items for Systematic Reviews and Meta-Analyses (PRISMA) guidelines (18). We searched PubMed, Embase, Cochrane Library, Web of Science, China National Knowledge Infrastructure (CNKI), Wanfang Database, Chinese Scientific Journal Database (VIP), and Chinese Biomedical Literature Database (CBM) databases from inception to 23 June 2023 and identified randomized controlled trials (RCTs) examining the use of acupuncture in patients experiencing fatigue, depression, anxiety, cognitive dysfunction, headache, and insomnia, provided these symptoms were not caused by other chronic conditions. The selection of these symptoms was based on published studies that reported the common symptoms of long COVID (8, 9, 19, 20). We also carried out backward and forward searching to identify additional studies from citations. The completed search formula for each database is depicted in Supplementary Table 1.

### 2.2 Study selection

Detailed inclusion and exclusion criteria are listed in Supplementary Table 2. RCTs reporting patients with at least one of the targeted neurological or neuropsychiatric symptoms (fatigue, depression, anxiety, cognitive abnormalities, headache, and insomnia) were included. Studies were excluded if the symptoms were caused by other chronic disorders.

Two pairs of reviewers (MXYL and YZ, DW and WCL) reviewed the title and abstract of each article and selected studies for a full-text review based on the inclusion and exclusion criteria. Conflicts were resolved through discussions and the involvement of a third researcher (LLZ).

### 2.3 Data extraction

Three reviewers (HL, WCL, and MXYL) extracted participant characteristic data from the selected studies using a standardized data extraction form. We extracted the following information from each included article: first author, year of publication, type and duration of the diseases, diagnostic criteria for the neurological and neuropsychiatric symptoms, number of participants, demographic data of participants, characteristics of the acupuncture and control groups, outcome data, duration of follow-ups, number of dropouts, and reports of adverse effects. Given that the number of participants and dropouts might vary at different stages in the same study, we considered the sample size included in the intention-to-treat analysis and the number of dropouts at the end of interventions.

### 2.4 Risk of bias assessment

All reviewers underwent comprehensive training for conducting assessments using the guidance document developed by the Cochrane Risk-of-Bias Tool for Randomized Trials (RoB 2) Development Group (21). The training encompassed a detailed understanding of the assessment process, including the nuances of each bias domain and the application of the tool's criteria. Two reviewers (DW and MXYL) assessed the potential risk of bias for all included studies in five different domains: (1) bias arising from the randomization process; (2) bias due to deviations from intended interventions; (3) bias due to missing outcome data; (4) bias in the measurement of the outcome; and (5) bias in the selection of the reported result. Each domain was assessed for risk of bias as “low risk,” “some concerns,” or “high risk,” and an overall consensus about the risk of bias was reached. In cases where a high risk of bias was identified, detailed documentation of the particular concerns within each domain was undertaken. A third review author (WCL) acted as an adjudicator in the event of disagreement.

### 2.5 Statistical analysis

We conducted a systematic review and meta-analysis for the following symptoms: fatigue, depression, anxiety, mild cognitive impairment, headache, and insomnia. For the meta-analysis, mean differences (MDs) with 95% confidence intervals (CIs) were adopted as the effect size for continuous outcomes. Forest plots were generated to visually assess the effect size, corresponding 95% CIs, and heterogeneity. Heterogeneity was assessed by conducting the chi-squared test and I^2^. The values of I^2^ <25%, 25–50%, and >50% indicated low, moderate, and high heterogeneity, respectively. A random model was used if there was significant heterogeneity (I^2^ >50%); otherwise, a fixed model was used. Subgroup analyses were based on the type of acupuncture (electro-acupuncture or manual acupuncture), treatment duration (short-term: ≤ 4 weeks or long-term: >4 weeks), and the type of medication. Sensitivity analyses were performed by limiting our analyses to high-quality studies to determine the robustness of the meta-analysis. Funnel plots were utilized, and Begg's test was conducted to detect the publication bias when at least 10 studies were reporting the outcome(s). The statistical analysis was performed by a statistician (LHJ) using R software, version 4.3.2.

## 3 Results

### 3.1 Search results

**Figure 1:**
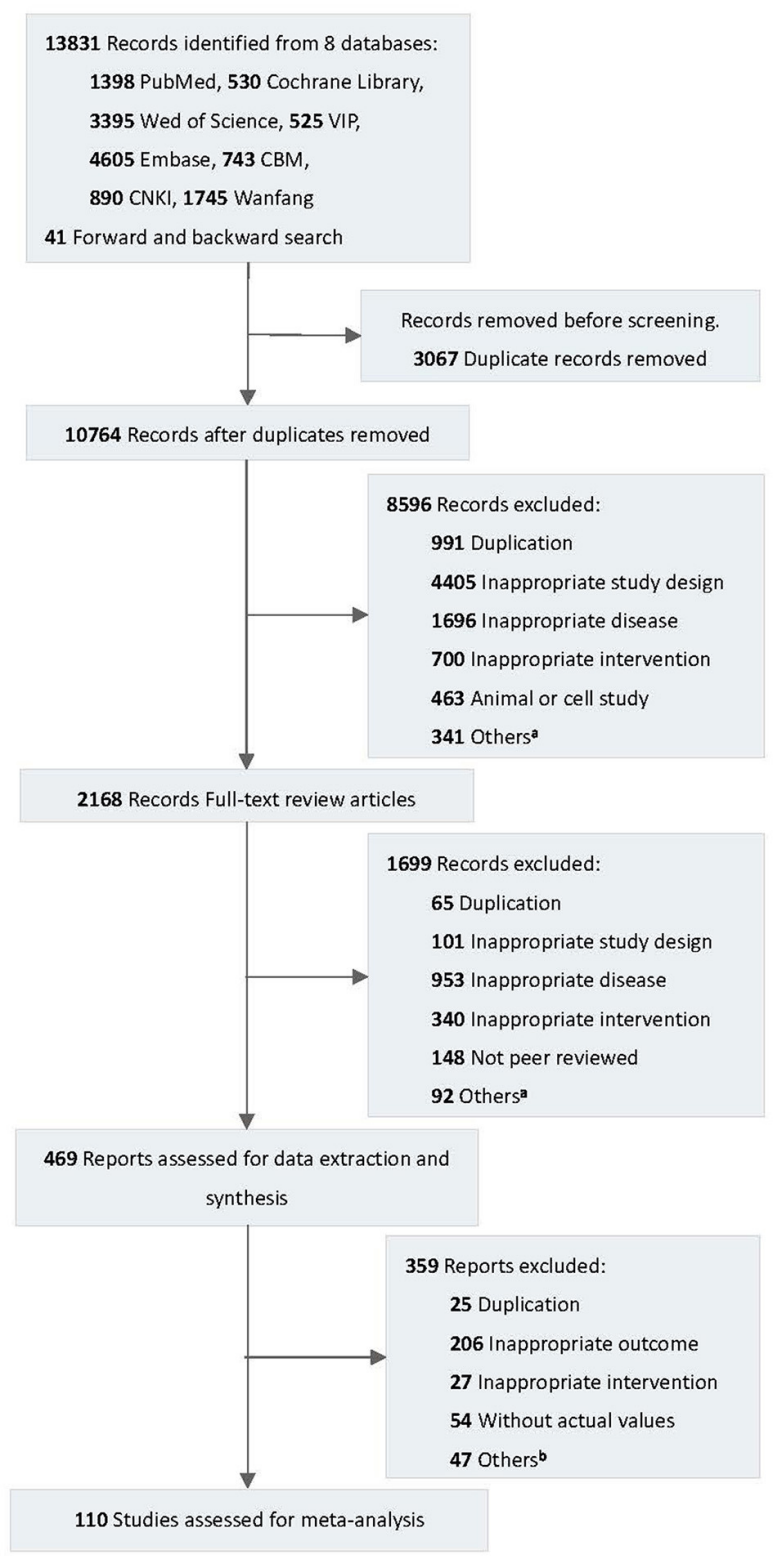
Flow diagram of the study selection process. VIP, Chinese Scientific Journal Database; CBM, Chinese Biomedical Literature Database; CNKI, China National Knowledge Infrastructure. ^a^Includes protocol, conference abstract, etc. ^b^Includes non-peered reviewed articles, incomparable type of data, etc.

A total of 13,831 records were identified from eight databases (Figure 1). After excluding duplicate records, 10,764 records were screened. A total of 2,168 full-text records were assessed for eligibility through preliminary screening. Finally, 110 studies met the inclusion and exclusion criteria and were included in our review and quantitative synthesis.

### 3.2 Study characteristics

Out of 110 RCTs included in our study, the sample sizes ranged from 30 to 440. A total of 11 studies specifically concerned fatigue, 32 concerned depression or anxiety, 7 concerned mild cognitive impairment, 18 concerned headache, and 42 concerned insomnia. The overall treatment duration varied from 10 days to 4 months. Most of the trials were two-armed, with the exception of one four-armed trial and 14 three-armed trials. The detailed characteristics of each study are presented in Supplementary Table 3.

### 3.3 Risk of bias in included studies

**Figure 2:**
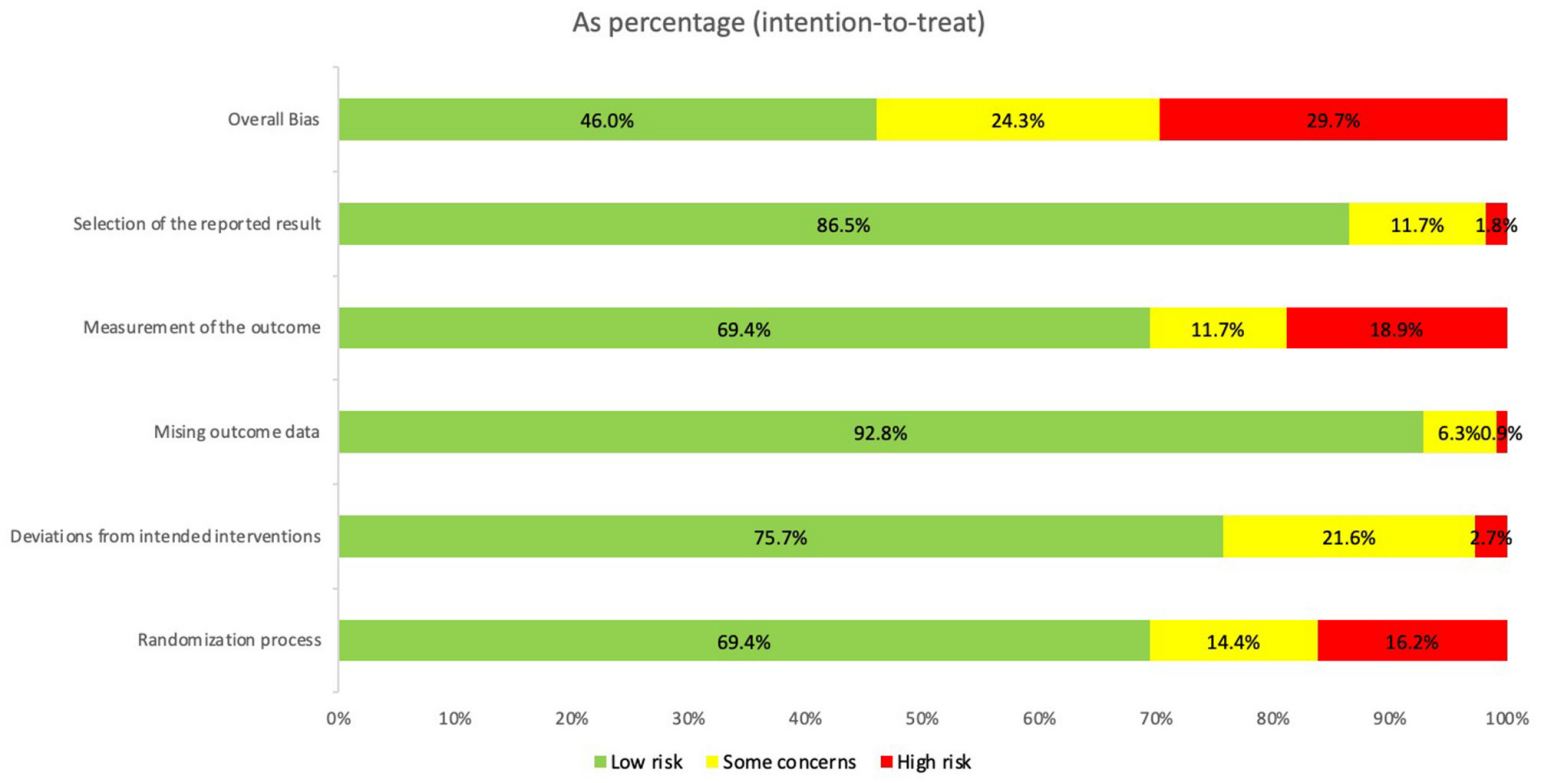
Risk of bias assessment of included studies.

Figure 2 shows the overall risk of bias, and the details of each study are shown in Supplementary Figure 1. A total of 79 out of 110 studies reported the randomization process. Approximately 84% of the 79 articles specified the generation of random sequences and 82% stated the allocation concealment. A total of 31 studies were rated as having a high risk of performance bias or unclear performance bias (blinding of participants and personnel). Out of these 79 studies, 64 performed single blinding of patients by using true and sham techniques for stimulating acupoints, and 59 studies reported using blinded assessors. No study was double-blinded as it is difficult to blind the acupuncture therapist. Other biases were caused by unreported dropouts or a large number of dropouts affecting the results (22–27), a change in protocol during the course of the trial (22, 28–35), potential carryover effects in crossover studies (23, 25, 26, 28, 33, 35–50), and problems associated with the statistical analysis of results and the tendency to draw optimistic conclusions (25, 26, 28, 35, 36, 38, 42, 43, 47, 51–55). In conclusion, the assessment of the risk of bias highlights crucial methodological considerations. These revelations necessitate meticulous consideration when interpreting the outcomes of the meta-analysis.

### 3.4 Results of the meta-analysis

**Figure 3:**
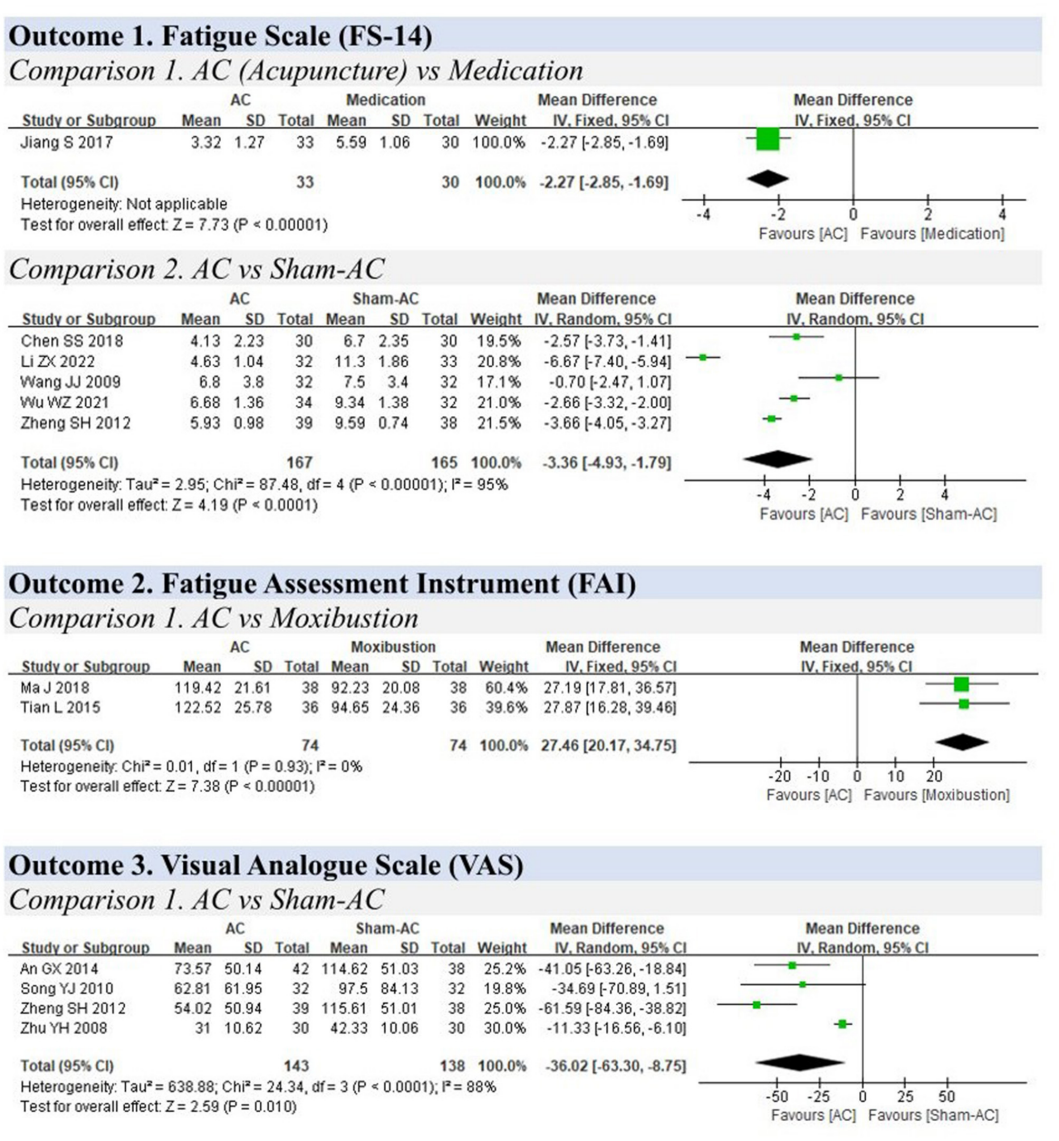
Forest plots for fatigue.

**Figure 4:**
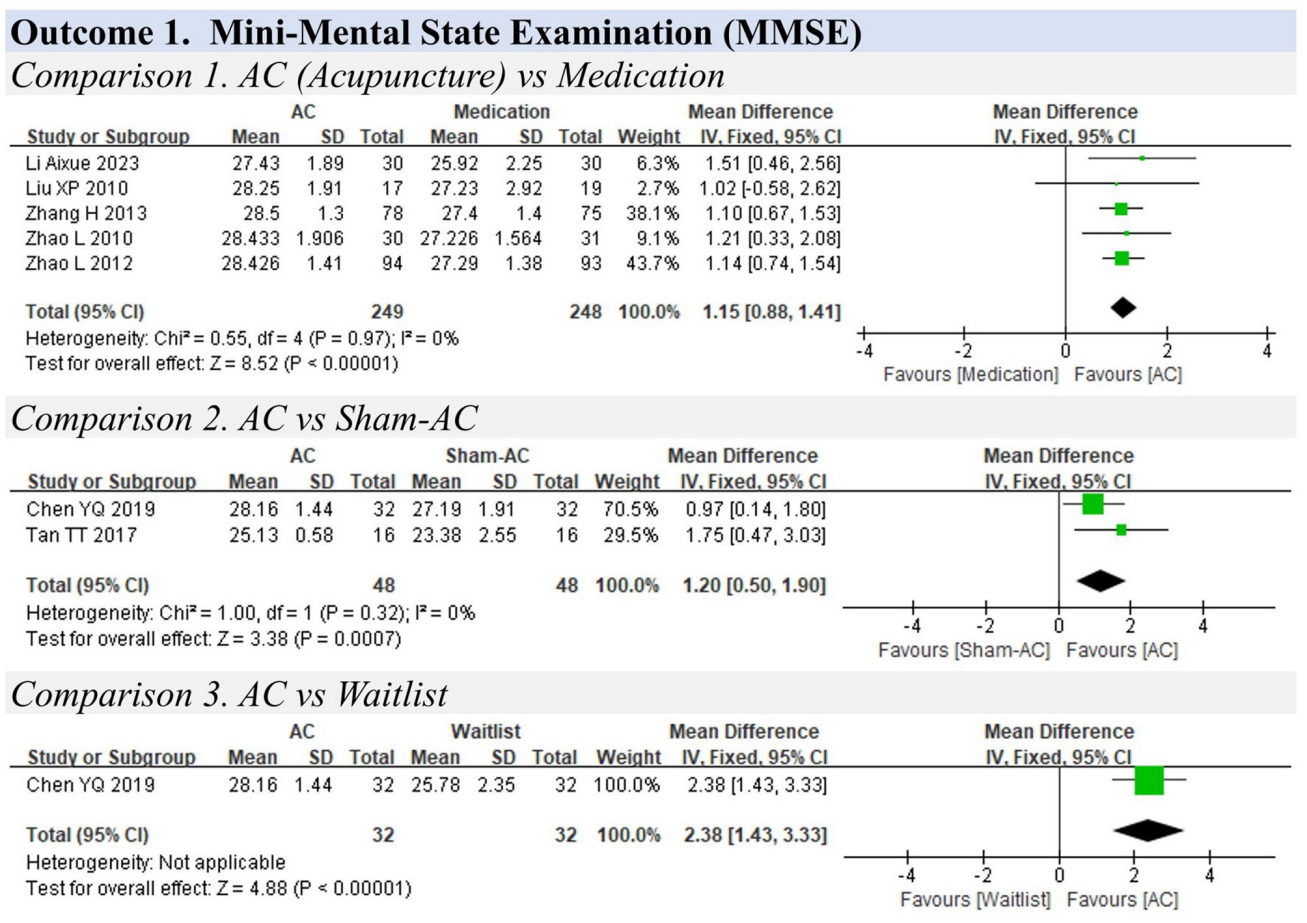
Forest plots for depression and anxiety.

**Figure 5:**
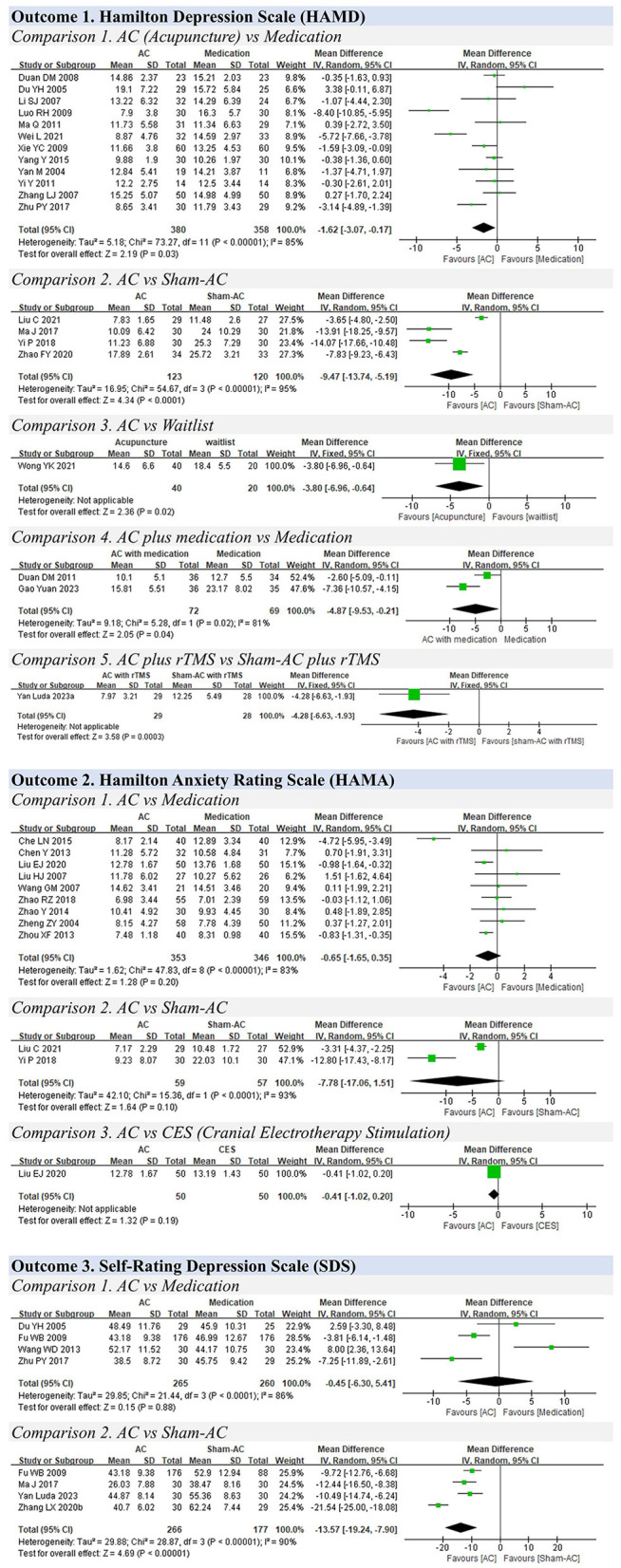
Forest plots for mild cognitive impairment.

**Figure 6:**
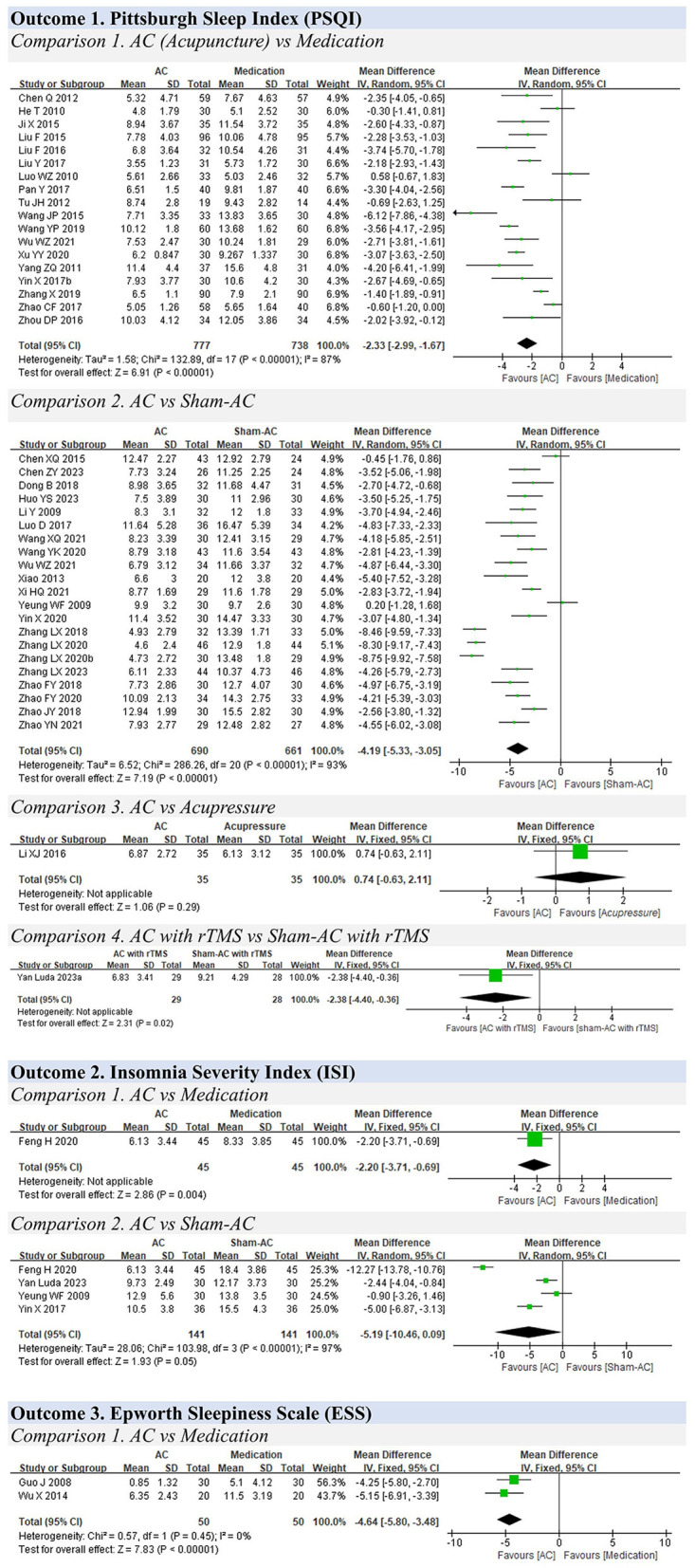
Forest plots for headache.

**Figure 7:**
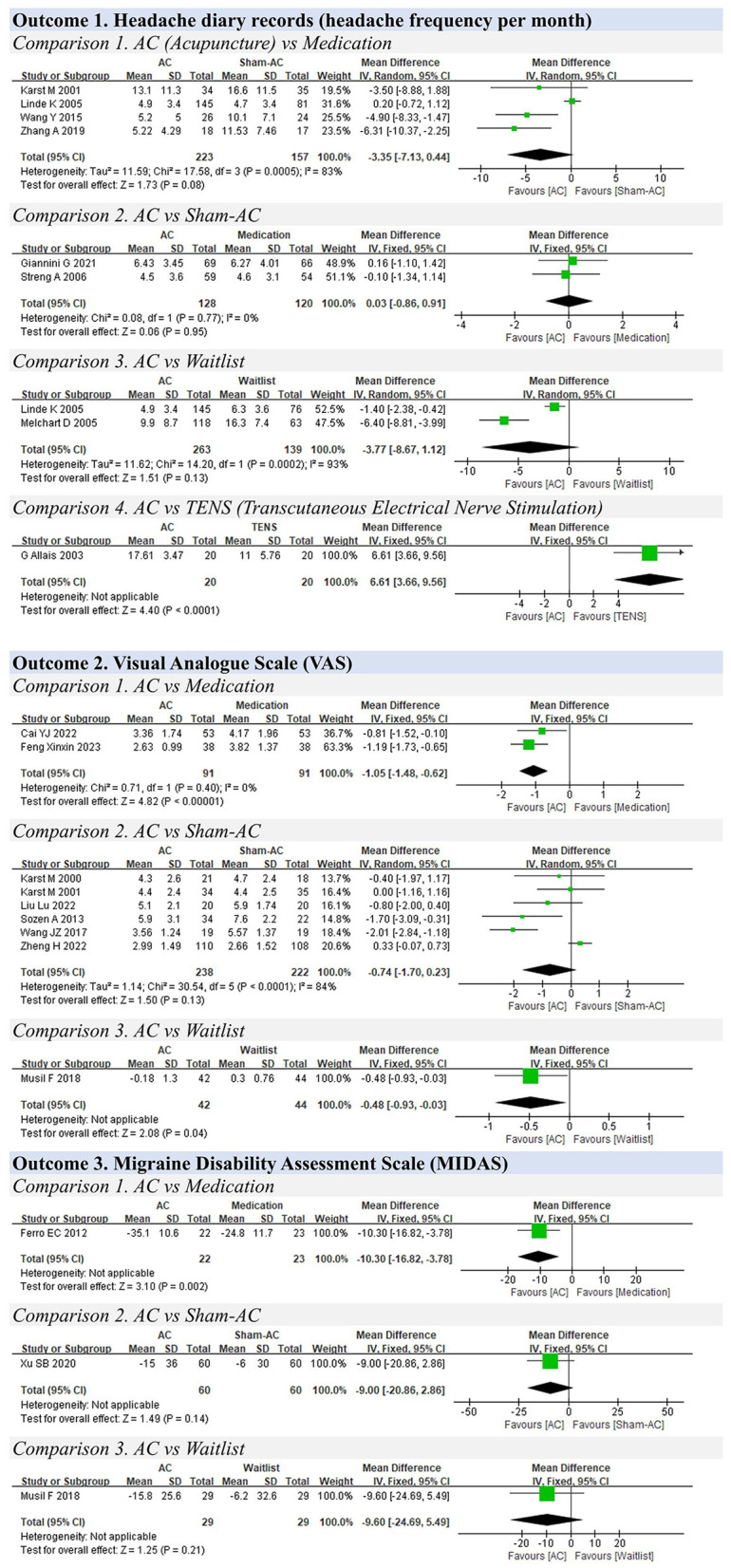
Forest plots for insomnia.

A total of 110 studies with 8,514 participants were included in the meta-analysis. The results of the meta-analysis are presented in Table 1. The forest plots of the meta-analysis are shown in Figures 3–7.

**Table 1:** The meta-analysis results.

	**Studies**	**Participants**	**Model**	**MD, 95% CIs**	**I^2^, %**
**Fatigue**					
***FS-14***					
AC vs. medication	1	63	Fixed	−2.27 [−2.85, −1.69]^*^	0
AC vs. Sham AC	5	332	Random	−3.36 [−4.91, −1.79]^*^	95
***FAI***					
AC vs. moxibustion	2	148	Fixed	27.46 [20.17, 34.75]^*^	0
***VAS***					
AC vs. Sham AC	4	281	Random	−36.02 [−63.30, −8.75]^*^	88
**Depression and anxiety**					
***HAMD***					
AC vs. medication	12	738	Random	−1.62 [−3.07, −0.17]^*^	85
AC vs. sham AC	4	243	Random	−9.47 [−13.74, −5.19]^*^	95
AC vs. waitlist	1	60	Fixed	−3.80 [−6.96, −0.64]^*^	0
AC plus medication vs. medication	2	141	Fixed	−4.87 [−9.53, −0.21]^*^	35
AC plus rTMS vs. Sham AC plus rTMS	1	57	Fixed	−4.28 [−6.63, −1.93]^*^	0
***HAMA***					
AC vs. medication	9	961	Random	−0.65 [−1.65, 0.35]	83
AC vs. Sham AC	2	116	Random	−7.78 [−17.06, 1.51]	93
AC vs. CES	1	100	Fixed	−0.41 [−1.02, 0.20]	0
***Assessed by the SDS***					
AC vs. medication	4	525	Random	−0.45 [−6.30, 5.41]	86
AC vs. Sham AC	4	443	Random	−13.57 [−19.24, −7.90]^*^	90
**Cognitive impairment**					
***MMSE***					
AC vs. medication	5	497	Fixed	1.15 [0.88, 1.41]^*^	0
AC vs. Sham AC	2	96	Fixed	1.20 [0.50, 1.90]^*^	0
AC vs. waitlist	1	64	Fixed	2.38 [1.43, 3.33]^*^	0
**Headache**					
***Headache diary records (mean frequency per month)***					
AC vs. medication	2	248	Random	0.03 [−0.86, 0.91]	0
AC vs. Sham AC	4	380	Random	−3.35 [−7.13, 0.44]	83
AC vs. waitlist	2	402	Random	−3.77 [−8.67, 1.12]	93
AC vs. TENS	1	40	Fixed	6.61 [3.66, 9.56]^*^	0
***VAS***					
AC vs. medication	2	182	Fixed	−1.05 [−1.48, −0.62]^*^	0
AC vs. Sham AC	6	460	Random	−0.74 [−1.70, 0.23]	84
AC vs. waitlist	1	86	Fixed	−0.48 [−0.93, −0.03]^*^	0
***MIDAS***					
AC vs. medication	1	45	Fixed	−10.30 [−16.82, −3.78]^*^	0
AC vs. Sham AC	1	120	Fixed	−9.00 [−20.86, 2.86]	0
AC vs. waitlist	1	58	Fixed	−9.60 [−24.69, 5.49]	0
**Insomnia**					
***PSQI***					
AC vs. medication	18	1515	Random	−2.33 [−2.99, −1.67]^*^	87
AC vs. Sham AC	21	1351	Random	−4.19 [−5.33, −3.05]^*^	93
AC vs. acupressure	1	70	Fixed	0.74 [−0.63, 2.11]	0
AC plus rTMS vs. Sham AC plus rTMS	1	57	Fixed	−2.38 [−4.40, −0.36]^*^	0
***ISI***					
AC vs. medication	1	90	Fixed	−2.20 [−3.71, −0.69]^*^	0
AC vs. Sham AC	4	282	Random	−5.19 [−10.46, 0.09]	97
***ESS***					
AC vs. medication	2	100	Fixed	−4.64 [−5.80, −3.48]^*^	0

^*^statistically significant

AC, Acupuncture; CES, Cranial Electrotherapy Stimulation; ESS, Epworth Sleepiness Scale; FAI, Fatigue Assessment Instrument; FS-14, Fatigue Scale; HAMA, Hamilton Anxiety Rating Scale; HAMD, Hamilton Depression Rating Scale; ISI, Insomnia Severity Index; MIDAS, Migraine Disability Assessment; MMSE, Mini-Mental State Examination; PSQI, Pittsburgh Sleep Quality Index; rTMS, Repetitive Transcranial Magnetic Stimulation; SDS, Self-Rating Depression Scale; TENS, Transcutaneous Electrical Nerve Stimulation; VAS, Visual Analog Scale.

### 3.5 Fatigue

**Outcome 1. Fatigue Scale (FS-14)**

**Comparison 1. Acupuncture vs. medication**

One study compared acupuncture and medication for the treatment of fatigue as measured using the FS-14 (0–14 scores) and found that acupuncture could relieve the symptoms of fatigue (MD: −2.27, 95% CIs: −2.85 to −1.69, *P* < 0.01) (Figure 3) (56).

**Comparison 2. Acupuncture vs. sham acupuncture**

Five trials that compared acupuncture with sham acupuncture were included (57–61). The result indicated that acupuncture could reduce the scores of the FS−14 and improve fatigue symptoms compared to sham acupuncture (MD: −3.36, 95% CIs: −4.91 to −1.79, *P* < 0.01), although heterogeneity was present (I^2^ = 95%).

**Outcome 2. Fatigue Assessment Instrument (FAI)**

Two studies compared acupuncture with moxibustion (62, 63). The pooled analysis indicated that acupuncture is inferior to moxibustion in treating fatigue symptoms (MD: 27.46, 95% CIs: 20.17 to 34.75, I^2^ = 0%, *P* < 0.01).

**Outcome 3. Visual Analog Scale (VAS)**

Four studies compared acupuncture with sham acupuncture (59, 64–66), and the results showed that acupuncture could decrease chronic fatigue–related pain (MD: −36.02, 95% CIs: −63.30 to −8.75, I^2^ = 88%, *P* = 0.01).

### 3.6 Depression and anxiety

**Outcome 1. Hamilton Depression Rating Scale (HAMD)**

**Comparison 1. Acupuncture vs. medication**

Twelve studies involving 738 participants were included in this pooled analysis (28, 36–38, 51, 52, 67–72). The results showed that acupuncture was better than medication in improving depressive symptoms (MD: −1.62, 95% CIs: −3.07 to −0.17, *P* < 0.01). However, there was substantial heterogeneity among these studies (I^2^ = 85%) (Figure 4).

The subgroup analysis based on treatment duration (≤ 4 weeks vs. >4 weeks) indicated that short–term efficacy of acupuncture was better (≤ 4 weeks: MD: −2.96, 95% CIs: −5.47 to −0.45; >4 weeks: MD: −0.68, 95% CIs: −2.40 to 1.03) than medication. The subgroup analysis of electro–acupuncture or manual acupuncture failed to show significant benefits for treating depression compared with medication (electro–acupuncture: MD: −0.89, 95% CIs: −1.82 to 0.05; manual acupuncture: MD: −1.80, 95% CIs: −3.84 to 0.24) (Supplementary Figure 2). However, there was heterogeneity among the subgroups of ≤ 4 weeks, >4 weeks, and manual acupuncture.

A sensitivity analysis was conducted on the remaining high–quality studies assessed by the RoB 2 Development Group to investigate the influence of study quality on the effect size. The result of the sensitivity analysis showed that acupuncture was not superior to medication (fluoxetine) in treating depression (MD: −1.32, 95% CIs: −6.23, 3.59; P <0.01). However, the heterogeneity was still significant (I^2^ = 93%) (Supplementary Figure 3).

**Comparison 2. Acupuncture vs. sham acupuncture**

In four studies (73–76), acupuncture was shown to have better effectiveness than sham acupuncture in relieving depressive symptoms (MD: −9.47, 95% CIs: −13.74 to −5.19, I^2^=95%, *P* < 0.01).

**Comparison 3. Acupuncture vs. waitlist**

One study reported that acupuncture was more effective in relieving depressive symptoms than the control waitlist group (MD: −3.80, 95% CIs: −6.96 to −0.64, *P* = 0.02) (77).

**Comparison 4. Acupuncture plus medication vs. medication**

Two studies reported that acupuncture with medication was more effective in relieving depressive symptoms than medication alone (MD: −4.87, 95% CIs: −9.53 to −0.21, I^2^ = 35%, *P* < 0.01) (78, 79).

**Comparison 5. Acupuncture plus rTMS vs. sham acupuncture plus rTMS**

One study compared acupuncture plus Repetitive Transcranial Magnetic Stimulation (rTMS) with sham acupuncture plus rTMS (80). The acupuncture plus rTMS group demonstrated better efficacy (MD: −4.28, 95% CIs: −6.63, −1.93, *P* < 0.01).

**Outcome 2. Hamilton Anxiety Rating Scale (HAM–A)**

Nine studies compared acupuncture with medication using the HAM–A scores (30–35, 39, 81, 82). These studies did not show any significant difference between the two groups (MD: −0.65, 95% CIs: −1.65, 0.35, I^2^ = 83%, *P* < 0.01).

Two studies compared acupuncture with sham acupuncture and did not show any significant difference between the two groups (MD: −7.78, 95% CIs: −17.06, 1.51, I^2^ = 93%, *P* = 0.10) (73, 75).

One study compared acupuncture with Cranial Electrotherapy Stimulation (CES) (82). There was no significant difference between the two groups (MD: −0.41, 95% CIs: −1.02, 0.20, *P* = 0.19).

**Outcome 3. Self–Rating Depression Scale (SDS)**

Four studies compared acupuncture with medication using the SDS and found no significant difference between the two groups (MD: −0.45, 95% CIs: −6.30, 5.41, I^2^ = 86%, *P* = 0.88) (22, 29, 38, 70).

Four studies compared acupuncture with sham acupuncture, and it was found that acupuncture could reduce the scores of the SDS (MD: −13.57, 95% CIs: −19.24, −7.90, I^2^ = 90%, *P* < 0.01) (29, 74, 83, 84).

### 3.7 Mild cognitive impairment

**Outcome 1. Mini-Mental State Examination (MMSE)**

**Comparison 1. Acupuncture vs. medication**

Five studies compared acupuncture with medication using the MMSE (ranging from 0 to 30) (23, 85–88) and showed that acupuncture was slightly better than medication in improving mild cognitive impairment (MD: 1.15, 95% CIs: 0.88 to 1.41, *P* < 0.01), without significant heterogeneity (I^2^ = 0%) (Figure 5).

**Comparison 2. Acupuncture vs. sham acupuncture**

A pooled analysis of two studies indicated that acupuncture could slightly relieve the symptoms of mild cognitive impairment compared with sham acupuncture (MD: 1.20, 95% CIs: 0.50 to 1.90, I^2^=0%, P <0.01) (53, 89).

**Comparison 3. Acupuncture vs. waitlist**

One study indicated that acupuncture could relieve cognitive impairment symptoms compared with the waitlist (MD: 2.38, 95% CIs: 1.43 to 3.33, *P* < 0.01) (89).

### 3.8 Headache

**Outcome 1. Headache diary records (headache frequency per month)**

Two studies compared the overall scores between acupuncture and medication (24, 90), and no significant difference was reported (MD: 0.03, 95% CIs: −0.86 to 0.91, I^2^ = 0%, *P* = 0.95) (Figure 6).

Four studies compared acupuncture with sham acupuncture (54, 91–93), and no obvious difference was found between the two groups (MD: −3.35, 95% CIs: −7.13 to 0.44, I^2^ = 83%, *P* = 0.08).

Two studies compared acupuncture with the waitlist control (93, 94), and no significant difference was detected (MD: −3.77, 95% CIs: −8.67 to 1.12, I^2^ = 93%, *P* = 0.13).

One study compared acupuncture with Transcutaneous Electrical Nerve Stimulation (TENS) (95), and it showed that TENS was better than acupuncture for relieving headache (MD: 6.61, 95% CIs: 3.66 to 9.56, *P* < 0.01).

**Outcome 2. VAS**

**Comparison 1. Acupuncture vs. medication**

Two studies compared acupuncture with medication using the VAS scores for headache. The results showed that acupuncture is slightly better than medication therapy in reducing headache (MD: −1.05, 95% CIs: −1.48 to −0.62, I^2^ = 0%, P <0.01) (55, 96).

**Comparison 2. Acupuncture vs. sham acupuncture**

Six studies compared acupuncture with sham acupuncture (40, 54, 97–100). The results showed that acupuncture was not superior to sham acupuncture for relieving headache (MD: −0.74, 95% CIs: −1.70 to 0.23, I^2^ = 84%, *P* = 0.13).

**Comparison 3. Acupuncture vs. waitlist**

One study compared acupuncture with waitlist control (101). It showed a slight difference between the two groups (MD: −0.48, 95% CIs: −0.93 to −0.03, *P* = 0.04).

**Outcome 3. Migraine Disability Assessment Scale (MIDAS)**

One study compared acupuncture with medication using the MIDAS and showed that acupuncture could reduce the overall scores after treatment compared with medication (MD: −10.30, 95% CIs: −16.82 to −3.78, P <0.01) (102).

One study compared acupuncture with sham acupuncture (103). There was no significant difference reported between the two groups (MD: −9.00, 95% CIs: −20.86 to 2.86, *P* = 0.14).

One study compared acupuncture with the waitlist (101). It failed to show any significant difference between the two groups (MD: −9.60, 95% CIs: −24.69 to 5.49, *P* = 0.21).

### 3.9 Insomnia

**Outcome 1. Pittsburgh Sleep Quality Index (PSQI)**

**Comparison 1. Acupuncture vs. medication**

A total of 18 studies were included in the analysis of insomnia measured using the PSQI (0–21 scores) (25, 26, 41–46, 48, 104–112). Acupuncture was reported to have better efficacy for insomnia than medication (MD: −2.33, 95% CIs: −2.99 to −1.67, I^2^ = 87%, *P* < 0.01) (Figure 7).

The subgroup analysis showed that there was a slight difference between the electro–acupuncture and manual acupuncture groups (electro–acupuncture: MD: −2.71, 95% CIs: −3.81 to −1.61; manual acupuncture: MD: −2.31, 95% CIs: −3.00 to −1.61; P =0.54) (Supplementary Figure 2). In addition, alprazolam showed better efficacy than other medications. A second subgroup analysis was not conducted in the present study because the treatment duration of all included studies was <4 weeks.

A sensitivity analysis was carried out exclusively on studies with a low risk of bias. Among six high–quality studies, it was shown that acupuncture provided a slight benefit for treating insomnia compared with medication (estazolam) (MD: −3.44, 95% CI: −4.39, −2.50, *P* < 0.01). This result was consistent with the findings of the total meta–analysis (Supplementary Figure 3).

**Comparison 2. Acupuncture vs. sham acupuncture**

A total of 21 studies compared the efficacy of acupuncture with that of sham acupuncture (27, 60, 76, 84, 113–129). The overall meta–analysis result showed that acupuncture had better efficacy for treating insomnia (MD: −4.19, 95% CIs: −5.33 to −3.05, I^2^=93%, *P* < 0.01). The results of the two subgroup analyses indicated that manual acupuncture had better efficacy than electro–acupuncture, and acupuncture was found to be more effective in the short term than in the long term (Supplementary Figure 2).

The sensitivity analyses solely included high–quality studies, which showed that acupuncture was beneficial for treating insomnia compared with sham acupuncture (MD: −4.36, 95% CI: −5.22, −3.50, *P* < 0.01). The finding was consistent with the result of the meta–analysis (Supplementary Figure 3).

**Comparison 3. Acupuncture vs. non–invasive intervention**

One study compared acupuncture with acupressure (49). There was no difference between the two groups (MD: 0.74, 95% CIs: −0.63 to 2.11, *P* = 0.29).

**Comparison 4. Acupuncture plus rTMS vs. sham acupuncture plus rTMS**

One study compared acupuncture plus rTMS with sham acupuncture plus rTMS (80). The acupuncture plus rTMS group showed better efficacy (MD: −2.38, 95% CIs: −4.40, −0.36, *P* = 0.02).

**Outcome 2. Insomnia Severity Index (ISI)**

One study compared acupuncture with medication using the ISI and showed that acupuncture could reduce the overall scores after treatment compared with medication (MD: −2.20, 95% CIs: −3.71 to −0.69, P <0.01) (130).

Four studies compared acupuncture with sham acupuncture. The acupuncture group did not show better efficacy (MD: −5.19, 95% CIs: −10.46 to 0.09, I^2^ = 97%, *P* = 0.05) (83, 122, 130, 131).

**Outcome 3. Score changes measured using the Epworth Sleepiness Scale (ESS)**

Two studies compared acupuncture with medication using the ESS and showed that acupuncture could reduce the overall scores compared with medication (MD: −4.64, 95% CIs: −5.80 to −3.48, I^2^ = 0%, *P* < 0.01) (47, 50).

### 3.10 Adverse effects

A total of 45 out of 110 (40.91%) included studies reported that patients with adverse effects were observed in both acupuncture and control groups (Supplementary Table 3). Among the 2,917 patients included in the 45 studies, 227 (7.78%) experienced adverse effects during acupuncture treatment, including instances of local bleeding, hematoma, needling pain, and dizziness. These effects were generally mild in severity, and the patients recovered well after rest or simple treatment. A total of 115 patients out of 1,153 (9.97%) taking Western medication reported adverse effects. It was reported that the incidence of acupuncture-related adverse effects was not significantly higher than that of medication treatment. A total of 14 patients out of 2,917 (0.48%) receiving acupuncture treatment withdrew from the clinical trial due to intolerance to acupuncture or adverse effects. A total of 9 patients out of 2,917 (0.31%) receiving acupuncture treatment reported severe adverse effects, but all of these effects were found to not be due to the intervention.

### 3.11 Publication bias

The Begg's tests did not show any significant difference, and the *p*-values were 0.6209 (acupuncture vs. medication for insomnia), 0.3021 (acupuncture vs. sham acupuncture for insomnia), and 0.6810 (acupuncture with medication for depression). The visual inspection of the funnel plots also did not reveal any substantial asymmetry in these comparisons (Supplementary Figure 4).

## 4 Discussion

Our systematic review is the first to assess the efficacy and safety of acupuncture for relieving neurological and neuropsychiatric symptoms that are common in long COVID patients. Our results showed that, compared to medication or sham acupuncture, acupuncture improves the symptoms of fatigue, depression, cognitive impairment, headache, and insomnia but not anxiety. In addition to statistical relevance, clinical significance should also be considered. For the scales with statistical significance, the improvement in scores was within specific thresholds, such as 5–10% from the baseline scores. However, clinicians are advised to take an overall clinical perspective by reviewing the relevant scales to understand the comprehensive impact on the patient's health (132).

Our study included 110 original studies involving 8,514 participants and used multiple clinical scales to assess the effects of acupuncture on target symptoms. Before the submission of our manuscript, an additional search across databases was conducted, and it failed to uncover any published clinical trials applying acupuncture to treat long COVID patients. By identifying and critically evaluating evidence from RCTs, we aim to provide comprehensive and the best available evidence for clinicians and decision-makers.

Previous meta-analyses have demonstrated consistent conclusions regarding the effectiveness of acupuncture in treating fatigue (133), depression (134), mild cognitive impairment (135), migraine (136), and insomnia (137). Our study refined the scope by adjusting the eligibility criteria to include long COVID patients. In patients with fatigue, compared with the control groups, both acupuncture and moxibustion revealed significant improvements in chronic fatigue and related pain symptoms. In patients with depression and cognitive impairment, weak significant effects in the HAMD and MMSE suggested the potential advantages of acupuncture compared to the medication, sham acupuncture, and blank groups. However, it is important to mention that the sensitivity analyses among high-quality studies demonstrated that acupuncture was not superior to fluoxetine in treating depression. In patients with headache, the pooled analysis results showed that acupuncture could be beneficial in alleviating the pain intensity of headaches but ineffective in reducing headache frequency. In our findings related to patients with insomnia, we found that acupuncture could be effective not only in lowering the PSQI and ISI scores but also in helping patients recover from “daytime sleepiness.”

There were certain differences in the analysis results among the subgroups based on various treatment durations, which might have been caused by different compliance rates and the severity of the disease. However, these differences were not significant. In addition to the comparison between short-term and long-term treatment effects, we also investigated whether there could be a significant difference in the treatment effects between manual acupuncture and electro-acupuncture. Theoretically, the effectiveness of manual acupuncture could vary largely depending on the technique of the acupuncturists, while electro-acupuncture provides relatively stable and standard stimulation at the acupoint. However, our results did not demonstrate major variations between the two approaches.

Despite obtaining favorable results with acupuncture, fewer clinical research studies have been conducted on long COVID patients. There are two case studies published by Michael et al. (138) and Robert et al. (139). These studies described the use of acupuncture for the treatment of long COVID and showed the potential benefit of acupuncture for patients with chronic long COVID symptoms. Although several published studies have evaluated various pharmacological and non-pharmacological interventions for treating neurological and neuropsychiatric symptoms of long COVID, including conventional medications (140, 141), synbiotics (142), herbal formulas (143, 144), and physical and mindfulness training (145, 146), a lack of consensus on specific treatment protocols for long COVID patients remains.

According to our safety data, acupuncture can be considered relatively safe, with minor adverse effects occurring in 1 out of 13 patients; these adverse effects are mild and subside after rest or simple treatment. This finding is similar to that of another systematic review, which reported an overall risk of 9.31%, suggesting that patients experience at least one adverse effect during a series of acupuncture treatments (147). The occurrence of adverse effects in patients receiving acupuncture treatment is slightly lower than that in patients taking Western medication, which can be taken into consideration when recommending respective treatments to patients.

During the COVID-19 epidemic, several versions of acupuncture guidance have been developed and issued by professional bodies for recovery from COVID-19 (148, 149). However, the recommendations were solely based on the traditional Chinese medicine theory of restoring qi and the normal functions of the organs. From a biological mechanism perspective, studies suggested that neurological and neuropsychiatric symptoms in long COVID patients could be caused by the devastating effects of direct viral neuro-invasion and/or systemic inflammation and cell-mediated immune mechanisms (150). Moreover, long-term social isolation with changes in living habits could potentially contribute to these symptoms. Acupuncture works through multiple mechanisms, including the alleviation of inflammation levels, the modulation of immune response, and the regulation of nitric oxide production for blood flow (151).

This review has several limitations that must be acknowledged. First, the original studies included in our systematic review did not specifically involve long COVID patients, which may limit the direct applicability of our findings. While we adjusted the eligibility criteria to reflect conditions similar to those experienced by long COVID patients, the absence of direct evidence from this specific group remains a significant constraint. Second, there were variations in the sample sizes, acupuncture protocols, and the clinical scales used to measure outcomes. Such variability can introduce biases and complicate the interpretation and generalizability of our findings. Third, our meta-analysis excluded studies with incomparable measures, which might have led to selection bias and limited our ability to comprehensively assess the efficacy of acupuncture across all potential outcome measures. Finally, the adverse effects in some original studies were underreported, which means that the safety profile of acupuncture might not have been completely captured. Our study has certain strengths, that is, the total sample size was large, it involved a comprehensive search strategy to identify and select studies without language restriction, and it included data across various age groups and disease durations.

## 5 Conclusion

This systematic review suggested acupuncture as a potentially beneficial approach for the treatment of neurological and neuropsychiatric symptoms associated with long COVID, as assessed using clinical scales, and it may have applicability in long COVID patients. Further well-designed clinical studies specifically targeting long COVID patients are needed to validate the role of acupuncture in alleviating long COVID symptoms.

## Data availability statement

The original contributions presented in the study are included in the article/Supplementary material, further inquiries can be directed to the corresponding authors.

## Author contributions

WL: Writing – original draft, Writing – review & editing. DW: Writing – original draft, Writing – review & editing. HL: Writing – original draft, Writing – review & editing. LY: Writing – review & editing. SZ: Writing – review & editing. ML: Writing – original draft, Writing – review & editing. YZ: Writing – review & editing. JY: Writing – review & editing. AYLL: Writing – review & editing. AL: Writing – review & editing. ZB: Writing – review & editing. AC: Writing – review & editing. LZ: Funding acquisition, Writing – review & editing.
