# Supplementary material for: The use of acupuncture for addressing neurological and neuropsychiatric symptoms in patients with long COVID: a systematic review and meta-analysis
Source: Front Neurol. 2024 Jul 19;15:1406475. doi: 10.3389/fneur.2024.1406475 (PMC11294104; doi:10.3389/fneur.2024.1406475)
Supplement: Supplementary file 1 [file Data_Sheet_1.docx]

**eTable 1. Search strategy**

**eTable 2. Inclusion/exclusion criteria**

**eTable 3. Characteristics of included studies**

**eTable 4. Adverse events summary**

**eTable 5. PRISMA 2020 Checklist**

**eFigure 1. Risk of bias assessment of included studies**

**eFigure 2. Subgroup analysis**

**eFigure 3. Sensitivity analysis**

**eFigure 4. Funnel plots**

**eTable 1. Search strategy**

| **1. PubMed**  (("fatigue"[MeSH Terms] OR "astheni*"[Title/Abstract] OR "weary"[Title/Abstract] OR "weariness"[Title/Abstract] OR "exhaustion"[Title/Abstract] OR "exhausted"[Title/Abstract] OR "lassitude"[Title/Abstract] OR "tired"[Title/Abstract] OR "frailty"[Title/Abstract] OR "drained"[Title/Abstract] OR "sleepy"[Title/Abstract] OR "sluggish"[Title/Abstract] OR "weak"[Title/Abstract] OR ("Anxiety"[MeSH Terms] OR "stress disorder"[Title/Abstract] OR "anxiety disorders"[Title/Abstract]) OR ("headache"[MeSH Terms] OR "cephalgia"[Title/Abstract] OR "cranial pain"[Title/Abstract]) OR "neurocognitive disorders"[MeSH Terms] OR ((("Confusion"[Title/Abstract] OR "cognit*"[Title/Abstract] OR "cognition"[Title/Abstract] OR "attention"[Title/Abstract] OR "memory"[Title/Abstract] OR "concentration"[Title/Abstract] OR "distract*"[Title/Abstract] OR "alert*"[Title/Abstract]) AND ("difficulties"[Title/Abstract] OR "Disorders"[Title/Abstract] OR "Disorder"[Title/Abstract] OR "issues"[Title/Abstract])) OR "brain fog"[Title/Abstract] OR "forgetfulness"[Title/Abstract] OR "brain dysfunction"[Title/Abstract] OR "cognitive dysfunction"[Title/Abstract] OR "cognitive impairment"[Title/Abstract] OR "neurocognitive decline"[Title/Abstract]) OR ("Anxiety"[MeSH Terms] OR "stress disorder"[Title/Abstract] OR "anxiety disorders"[Title/Abstract]) OR ("depressive disorder"[MeSH Terms] OR "depression"[MeSH Terms] OR "depressive disorder"[Title/Abstract] OR "affective disorder"[Title/Abstract] OR "affective symptoms"[Title/Abstract]) OR ("insomnia*"[MeSH Terms] OR "sleep disturbances"[Title/Abstract] OR "sleep*"[Title/Abstract] OR "sleep initiation"[Title/Abstract] OR "maintenance disorders"[Title/Abstract] OR "wakeful*"[Title/Abstract] OR "sleepless*"[Title/Abstract] OR "dyssomn*"[Title/Abstract] OR ("sleep-wake"[All Fields] AND "disordes"[Title/Abstract])) OR ("depressive disorder"[MeSH Terms] OR "depression"[MeSH Terms] OR "affective disorder"[Title/Abstract] OR "depressive disorder"[Title/Abstract]) OR "affective symptoms"[Title/Abstract]) AND ("acupuncture"[MeSH Terms] OR "acupuncture therapy"[MeSH Terms] OR ("electroacupuncture"[Title/Abstract] OR "electro-acupuncture"[Title/Abstract] OR "Manual Acupuncture"[Title/Abstract] OR "hand acupuncture"[Title/Abstract])) AND ("randomized controlled trial"[Publication Type] OR "controlled clinical trial"[Publication Type] OR "randomized"[Title/Abstract] OR "placebo"[Title/Abstract] OR "clinical trials as topic"[MeSH Terms:noexp] OR "randomly"[Title/Abstract] OR "trial"[Title])) NOT ("animals"[MeSH Terms] NOT ("humans"[MeSH Terms] AND "animals"[MeSH Terms])) |
| --- |
| **2. Web of Science**  (((((((((((((((TS=(fatigue)) OR TS=(astheni*)) OR TS=(weary)) OR TS=(weariness)) OR TS=(exhaustion)) OR TS=(exhausted)) OR TS=(lassitude)) OR TS=(tired)) OR TS=(frailty)) OR TS=(drained)) OR TS=(sleepy)) OR (TS=(sluggish)) OR TS=(weak))) OR ((TS=(Headache)) OR TS=(cephalgia)) OR TS=(cranial pain) OR (((((((((((((((((TS=(Neurocognitive disorders)) OR TS=(Confusion)) OR TS=(cognit*)) OR TS=(cognition)) OR TS=(memory)) OR TS=(concentration)) OR TS=(distract*)) OR TS=(alert*))) AND (TS=(difficulties)) OR TS=(disorders)) OR TS=(disorder)) OR TS=(issues))) OR TS=(Brain fog)) OR TS=(forgetfulness)) OR TS=(cognitive dysfunction)) OR TS=(brain dysfunction)) OR (TS=(cognitive impairment)) OR TS=(neurocognitive decline) OR ((((((((((TS=(insomnia*)) OR TS=(Sleep disturbances)) OR TS=(sleep*))OR TS=(Sleep Initiation)) OR TS=(Maintenance Disorders)) OR TS=(wakeful*)) OR TS=(sleepless*)) OR TS=(dyssomn*)) OR TS=(sleep-wake disordes) OR ((((TS=(depression) OR ((TS=(depressive disorder)) OR TS=(affective disorder)) OR TS=(affective symptoms) OR ((TS=(Anxiety)) OR TS=(stress disorder)) OR TS=(Anxiety disorders)  AND ((((TS=(acupuncture)) OR TS=(electroacupuncture)) OR TS=(electro-acupuncture)) OR TS=(Manual Acupuncture)) OR TS=(hand acupuncture)  AND (TS=(randomized controlled trial OR controlled clinical trial OR randomized OR placebo OR clinical trials OR randomly OR trial)) NOT (TS=(animals NOT (humans AND animals))) |
| **3. Cochrane Library**  #1 MeSH descriptor: [Fatigue] explode all trees  #2 (astheni* OR weary OR weariness OR exhaustion OR exhausted OR lassitude OR tired OR frailty OR drained OR sleepy OR sluggish OR weak):ti,ab,kw  #3 MeSH descriptor: [Anxiety] explode all trees  #4 (‘stress disorder’ OR ‘Anxiety Disorders’):ti,ab,kw  #5 MeSH descriptor: [Depression] explode all trees  #6 (‘Depressive Disorder’ OR ’affective disorder’ OR ‘affective symptoms’):ti,ab,kw  #7 MeSH descriptor: [Neurocognitive Disorders] explode all trees  #8 ((Confusion OR cognit* OR cognition OR attention OR memory OR concentration OR distract* OR alert*) and (difficulties OR disorders OR disorder OR issues) OR ‘brain fog’ OR forgetfulness OR ‘brain dysfunction’ OR ‘cognitive dysfunction’ OR ‘cognitive impairment’ OR ‘neurocognitive decline’):ti,ab,kw  #9 MeSH descriptor: [Headache] explode all trees  #10 ('cephalgia' OR 'cranial pain'):ti,ab,kw  #11 MeSH descriptor: [Sleep Initiation and Maintenance Disorders] explode all trees  #12 (insomnia* OR ‘Sleep disturbances’ OR sleep* OR ‘Sleep Initiation and Maintenance Disorders’ OR wakeful* OR sleepless* OR dyssomn* OR ‘sleep-wake disordes’):ti,ab,kw  #13 #1 OR #2 OR #3 OR #4 OR #5 OR #6 OR #7 OR #8 OR #9 OR #10 OR #11 OR #12  #14 MeSH descriptor: [Acupuncture] explode all trees  #15 ('manual acupuncture' OR 'hand acupuncture' OR 'electroacupuncture' OR 'electro-acupuncture'):ti,ab,kw  #16 #14 OR #15  #17 MeSH descriptor: [Randomized Controlled Trial] explode all trees  #18 ('randomised controlled trial' OR 'randomised controlled trial*' OR RCT* OR 'randomized controlled trial*' OR 'randomised-controlled trial'):ti,ab,kw  #19 (randomized controlled trials as Topic):ti,ab,kw  #20 (random allocation):ti,ab,kw  #21 (controlled clinical trials as Topic):ti,ab,kw  #22 (control groups):ti,ab,kw  #23 ('clinical trials as topic' or 'clinical trials, phase i as topic' or 'clinical trials, phase ii as topic' or 'clinical trials, phase iii as topic' or 'clinical trials, phase iv as topic'):ti,ab,kw  #24 (double-blind method):ti,ab,kw  #25 (single-blind method):ti,ab,kw  #26 (placebos):ti,ab,kw  #27 (placebo eGect):ti,ab,kw  #28 (cross-over studies):ti,ab,kw  #29 (randomized controlled trial):pt  #30 (controlled clinical trial):pt  #31 ((clinical trial or clinical trial phase i or clinical trial phase ii or clinical trial phase iii or clinical trial phase iv)):pt  #32 (animals not humans):ti,ab,kw  #33 #17 OR #18 OR #19 OR #20 OR #21 OR #22 OR #23 OR #24 OR #25 OR #26 OR #27 OR #28 OR #29 OR #30 OR #31 NOT #32  #34 #13 AND #16 AND #33 |
| **4. Embase**  #1 'fatigue'/exp OR 'fatigue'  #2 astheni* OR weary OR weariness OR exhaustion OR exhausted OR lassitude OR tired OR frailty OR drained OR sleepy OR sluggish OR weak:ab,kw,ti  #3 'anxiety'/exp  #4 'stress disorder' OR 'anxiety disorders':ab,kw,ti  #5 'depression'/exp  #6 'depressive disorder' OR 'affective disorder' OR 'affective symptoms':ab,kw,ti  #7 'neurocognitive disorders'/exp  #8 (confusion OR cognit* OR cognition OR attention OR memory OR concentration OR distract* OR alert*) AND (difficulties OR disorders OR disorder OR issues) OR 'brain fog' OR forgetfulness OR 'brain dysfunction' OR 'cognitive dysfunction' OR 'cognitive impairment' OR 'neurocognitive decline':ab,kw,ti  #9 'headache'/exp  #10 'cephalgia' OR 'cranial pain':ab,kw,ti  #11 'insomnia'/exp  #12 insomnia* OR 'sleep disturbances' OR sleep* OR 'sleep initiation and maintenance disorders' OR wakeful* OR sleepless* OR dyssomn* OR 'sleep-wake disordes':ab,kw,ti  #13 #1 OR #2 OR #3 OR #4 OR #5 OR #6 OR #7 OR #8 OR #9 OR #10 OR #11 OR #12  #14 'acupuncture'/exp  #15 'manual acupuncture' OR 'hand acupuncture' OR 'electroacupuncture' OR 'electro-acupuncture':ab,kw,ti  #16 #14 OR #15  #17 'crossover procedure':de OR 'double-blind procedure':de OR 'randomized controlled trial':de OR 'single-blind procedure':de OR random*:de,ab,ti OR factorial*:de,ab,ti OR crossover*:de,ab,ti OR ((cross NEXT/1 over*):de,ab,ti) OR placebo*:de,ab,ti OR ((doubl* NEAR/1 blind*):de,ab,ti) OR ((singl* NEAR/1 blind*):de,ab,ti) OR assign*:de,ab,ti OR allocat*:de,ab,ti OR volunteer*:de,ab,ti  #18 #13 AND #16 AND #17 |
| **5. China National Knowledge Infrastructure, CNKI**  (SU%=('虚劳' + '疲劳' + '疲倦' + '虚弱' + '乏力' + '头痛' + '注意力' + '认知' + '脑雾' + '头晕' + '焦虑'+ '紧张' + '焦躁' + '抑郁' + '忧郁' + '失眠' + '不寐' + '睡眠' + '嗜睡' + '意识' + '偏头痛' + '强迫症')) AND (SU%=('针灸' + '电针' + '针刺' + '手针' + '毫针')) AND (SU%=('随机对照')) |
| **6. Wanfang Database**  (主题:("虚劳" or "疲劳" or "疲倦" or "虚弱" or "乏力" or "头痛" or "注意力" or "认知" or "脑雾" or "头晕" or "焦虑" or "焦躁" or "抑郁" or "忧郁" or "失眠" or "不寐" or "睡眠" or "嗜睡" or "意识" or "偏头痛" or "紧张" or "强迫症")) AND (主题:("针灸" or "电针" or "针刺" or "手针" or "毫针")) AND (主题:("随机对照")) |
| **7. Chinese Scientific Journal Database (VIP)**  (((((((((((((((((((((((摘要=虚劳 OR 摘要=疲劳) OR 摘要=疲倦) OR 摘要=虚弱) OR 摘要=乏力) OR 摘要=焦虑) OR 摘要=焦躁) OR 摘要=抑郁) OR 摘要=忧郁) OR 摘要=紧张) OR 摘要=强迫症) OR 摘要=注意力) OR 摘要=认知) OR 摘要=脑雾) OR 摘要=意识) OR 摘要=痴呆) OR 摘要=头痛) OR 摘要=偏头痛) OR 摘要=失眠) OR 摘要=不寐) OR 摘要=睡眠) OR 摘要=嗜睡) AND 摘要=随机对照) AND ((((摘要=针灸 OR 摘要=电针) OR 摘要=针刺) OR 摘要=手针) OR 摘要=毫针)) |
| **8. Chinese Biomedical Literature Database (CBM)**  (( "针灸"[标题] OR "电针"[标题] OR "针刺"[标题] OR "手针"[标题] OR "毫针"[标题]) OR( "针灸"[摘要] OR "电针"[摘要] OR "针刺"[摘要] OR "手针"[摘要] OR "毫针"[摘要])) AND ("随机对照"[标题] OR "随机对照"[摘要]) AND (( "虚劳"[标题] OR "疲劳"[标题] OR "疲倦"[标题] OR "虚弱"[标题] OR "乏力"[标题] OR "焦虑"[标题] OR "焦躁"[标题] OR "抑郁"[标题] OR "忧郁"[标题] OR "紧张"[标题] OR "强迫症"[标题] OR "注意力"[标题] OR "认知"[标题] OR "脑雾"[标题] OR "意识"[标题] OR "痴呆"[标题] OR "头痛"[标题] OR "偏头痛"[标题] OR "失眠"[标题] OR "不寐"[标题] OR "睡眠"[标题] OR "嗜睡"[标题]) OR( "虚劳"[摘要] OR "疲劳"[摘要] OR "疲倦"[摘要] OR "虚弱"[摘要] OR "乏力"[摘要] OR "焦虑"[摘要] OR "焦躁"[摘要] OR "抑郁"[摘要] OR "忧郁"[摘要] OR "紧张"[摘要] OR "强迫症"[摘要] OR "注意力"[摘要] OR "认知"[摘要] OR "脑雾"[摘要] OR "意识"[摘要] OR "痴呆"[摘要] OR "头痛"[摘要] OR "偏头痛"[摘要] OR "失眠"[摘要] OR "不寐"[摘要] OR "睡眠"[摘要] OR "嗜睡"[摘要])) |

**eTable 2. Inclusion/exclusion criteria**

| Inclusion criteria:   1. Patients diagnosed with at least one of the targeted neurological or neuropsychiatric symptoms (fatigue, depression and/or anxiety, cognitive abnormalities, headache, and insomnia); 2. control groups with pharmacotherapy, sham acupuncture, waitlist, or other non-invasive interventions (include but are not limited to different types of cognitive behavioral therapy, exercise, homeopathy, music therapy, relaxation, tai chi, yoga, moxibustion, and massage); 3. studies reported widely adopted clinical outcome scales: for fatigue measured by Fatigue Scale (FS-14), Fatigue Assessment Instrument (FAI), and Visual Analogue Scale (VAS); for depression measured by Hamilton Depression Rating Scale (HAMD), Hamilton Anxiety Rating Scale (HAMA), and Self-Rating Depression Scale (SDS); for cognitive impairment measured by Mini-Mental State Examination (MMSE), and Activities of Daily Living (ADL); for headache measured by VAS, Headache Diary, and Migraine Disability Assessment (MIDAS); for insomnia measured by Pittsburgh Sleep Quality Index (PSQI), Insomnia Severity Index (ISI), and Epworth Sleepiness Scale (ESS); 4. randomized controlled trials (RCTs) published in peer-reviewed journals. |
| --- |
| Exclusion criteria:   1. Neurological or neuropsychiatric symptom(s) caused by other chronic disorders (for example, stroke, head trauma, epilepsy, tumor, or brain injury); 2. acupuncture type other than electro-acupuncture or manual acupuncture (for example, laser acupuncture); 3. self-control design; 4. total sample size less than 30. |

**eTable 3. Characteristics of included studies.**

| **ID** | **Author** | **Year** | **Target population** | **Diagnostic criteria** | **Sample size (A/C)** | **Gender (M/F)** | **Age^a^** | **Disease duration^a^** | **Type of A** | **Treatment Duration** | **Control group** | **Clinical outcome measures** | **Follow-up period** | **Dropout (A/C)** | **AE/SAE (Y/N/NI)** |
| --- | --- | --- | --- | --- | --- | --- | --- | --- | --- | --- | --- | --- | --- | --- | --- |
| 1^1^ | Wang, et al. | 2009 | Fatigue Syndrome | Clinical symptoms | 32/32 | 26/38 | A: 35.8 (10.7) C: 38.8 (8.8) | NI | Manual | 4 weeks | Sham A | FS | NA | 3/3 | N |
| 2^2^ | Chen, et al. | 2018 | Chronic Fatigue Syndrome | CDC-CFS | 30/30 | 20/40 | A: 40.8 (11.6) C: 41.5 (12.2) | A: 18.7 (9.2) months C: 17.8 (9.0) months | Manual | 4 weeks | Sham A | FS-14; SCL-90; Clinical Efficacy | NA | 0/0 | N |
| 3^3^ | Zheng, et al. | 2012 | Chronic Fatigue Syndrome | CDC-CFS | 39/38 | 31/46 | A: 38.7 (4.1) C: 37.1 (5.3) | A: 18.4 (5.3) months C: 17.1 (6.0) months | Manual | 4 weeks | Sham A | FS-14; VAS; DSI | NA | 1/2 | NI |
| 4^4^ | An, et al. | 2014 | Chronic Fatigue Syndrome | CDC-CFS | 42/38 | 35/45 | A: 36.5 (4.1) C: 37.1 (4.7) | NI | Manual | 4 weeks | Sham A | FS-14; VAS; DSI | NA | 0/0 | NI |
| 5^5^ | Wu, et al. | 2021 | Daytime Fatigue Insomnia | ICSD-3 | 34/32 | 26/40 | A: 47.0 (13.6) C: 46.2 (11.9) | A: 3.1 (4.2) years C: 2.8 (3.3) years | Electro | 4 weeks | Sham A | PSQI; FS-14 | 4 weeks | 1/3 | Y |
| 6^6^ | Ma, et al. | 2018 | Chronic Fatigue Syndrome | CDC-CFS | 38/38 | 36/40 | A: 43 (7) C: 43 (6) | NI | Manual | 40 days | Moxibustion | FAI; Clinical Efficacy | NA | 0/0 | N |
| 7^7^ | Tian, et al. | 2015 | Fatigue Syndrome | CDC-CFS | 36/36 | 40/32 | A: 42 (10) C: 42 (9) | NI | Manual | 30 days | Moxibustion | FAI; Clinical Efficacy | NA | 0/0 | N |
| 8^8^ | Song, et al. | 2010 | Chronic Fatigue Syndrome | CDC-CFS | 32/32 | 26/38 | A: 35.8 (10.7) C: 38.8 (8.8) | NI | Manual | 4 weeks | Sham A | VAS; Clinical Efficacy | NA | 3/3 | NI |
| 9^9^ | Zhu, et al. | 2008 | Chronic Fatigue Syndrome | CDC-CFS | 30/30 | 16/44 | A: 38.5 (7.9) C: 37.7 (9.9) | A: 15.3 (5.7) years C: 16.7 (5.4) years | Electro | 12 days | Sham A | FSS; SPHERE; VAS; SF-36 | NA | 0/0 | NI |
| 10^10^ | Jiang, et al. | 2017 | Chronic Fatigue Syndrome | CDC-CFS | 33/30 | 36/27 | O: 36.7 (4.1) | O: 4.6 (2.2) years | Manual | 120 days | Medication | FS-14; Clinical Efficacy | NA | 0/0 | NI |
| 11^11^ | Li, et al. | 2022 | Chronic Fatigue Syndrome | CDC-CFS | 36/36 | 28/37 | A: 35 (8) C: 36 (9) | A: 29.5 (14.2) months C: 29.0 (13.7) months | Electro | 6 weeks | Sham A | FS-14; SF-36 | NA | 4/3 | NI |
| 12^12^ | Li, et al. | 2007 | Melancholia | CCMD-3 | 32/24 | 25/31 | A: 49.2 (13.5) C: 47.0 (13.1) | A: 13.3 (12.8) months C: 9.5 (8.0) months | Electro | 6 weeks | Medication | HAMD; Clinical Efficacy | NA | 0/0 | NI |
| 13^13^ | Ma, et al. | 2011 | Depression | CCMD-3 | 31/29 | 24/36 | A: 51.1 (12.9) C: 50.9 (11.3) | A: 80.6 (40.3) days C: 78.9 (34.0) days | Manual | 6 weeks | Medication | HAMD; Clinical Efficacy; SERS | NA | 0/0 | Y |
| 14^14^ | Wei, et al. | 2021 | Depression | ICD-10 | 33/32 | 25/40 | A: 37.4 (11.4) C: 35.7 (12.2) | A: 4.7 (3.1) months C: 3.9 (4.2) months | Manual | 4 weeks | Medication | HAMD; PSQI; Clinical Efficacy | NA | 3/2 | Y |
| 15^15^ | Yang, et al. | 2015 | Depression | ICD-10 | 30/30 | 27/33 | A: 39.7 (13.4) C: 38.4 (12.2) | A: 3.2 (3.8) months C: 3.1 (4.3) months | Manual | 8 weeks | Medication | HAMD; Clinical Efficacy | 4 weeks | 0/0 | Y |
| 16^16^ | Xie, et al. | 2009 | Depression | ICD-10 | 60/60 | 44/76 | A: 41.7 (13.8) C: 42.4 (14.6) | A: 4.1 (3.5) years C: 5.0 (2.9) years | Manual | 8 weeks | Medication | HAMD; TESS | NA | 0/0 | Y |
| 17^17^ | Yan, et al. | 2004 | Depression | CCMD | 19/11 | 13/17 | A: 38 (5) C: 36 (8) | NI | Electro | 6 weeks | Medication | HAMD; CGIS | NA | 0/0 | NI |
| 18^18^ | Zhang, et al. | 2007 | Depression | CCMD-2-R | 50/50 | 26/74 | A: 25.7 (8.2) C: 26.1 (7.4) | NI | Manual | 4 weeks | Medication | HAMD | NA | 0/0 | NI |
| 19^19^ | Zhu, et al. | 2017 | Depression | CCMD-3 | 30/29 | 60/0 | A: 39.1 (13.8) C: 37.8 (12.8) | A: 5.5 (4.1) months C: 4.6 (3.7) months | Manual | 26 days | Medication | HAMD; SDS | NA | 0/1 | Y |
| 20^20^ | Duan, et al. | 2011 | Depression | ICD-10 | 36/34 | 26/49 | O: 35 (8) | O: 7.2 (2.4) months | Electro | 6 weeks | Medication | HAMD; Clinical Efficacy | NA | 2/3 | Y |
| 21^21^ | Luo, et al. | 2009 | Depression | CCMD-3 | 30/30 | 30/30 | A: 40 (4) C: 41 (5) | A: 2.1 (0.1) months C: 2.2 (0.2) months | Manual | 4 weeks | Medication | HAMD; Clinical Efficacy | NA | 0/0 | NI |
| 22^22^ | Du, et al. | 2005 | Depression | CCMD-3 | 29/25 | 21/39 | A: 42.8 (14.9) C: 43.9 (15.2) | A: 10.8 (11.3) months C: 9.7 (14.3) months | Manual | 6 weeks | Medication | HAMD; SDS; SCL-90; Clinical Efficacy | NA | 1/5 | Y |
| 23^23^ | Yi, et al. | 2011 | Depression | DSM-IV | 14/14/14 | 18/24 | A: 35.5 (7.4) C: 33.6 (8.4) A+C: 37.0 (8.6) | A: 8.0 (1.3) months C: 7.8 (1.4) months | Manual | 1 month | C1: Medication C2: Medication plus A | HAMD | NA | 0/0 | NI |
| 24^24^ | Duan, et al. | 2008 | Depression | CCMD-3 | 23/23/24 | 15/60 | A: 50.1 (4.3) C: 49.7 (5.5) A+C: 48.9 (7.6) | A: 16.2 (8.4) months C: 17.9 (4.8) months | Electro | 6 weeks | C1: Medication C2: Medication plus A | HAMD; Clinical Efficacy; TESS | NA | 2/2/1 | Y |
| 25^25^ | Liu, et al. | 2021 | Depression and Anxiety Chronic insomnia | ICSD-3 | 29/27 | 23/33 | A: 47.2 (14.1) C: 45.6 (12.7) | A: 2.7 (3.2) months C: 2.9 (3.6) months | Electro | 4 weeks | Sham A | PSQI; HAMA; HAMD | 3 months | 1/3 | Y |
| 26^26^ | Ma, et al. | 2017 | Depression | CCMD-3 DSM-IV | 30/30 | 37/23 | O: 48. 23 | O: 5.9 years | Manual | 8 weeks | Sham A | HAMD; SDS; TESS | 4 weeks | 0/2 | Y (TESS) |
| 27^27^ | Yin, et al. | 2018 | Depression | CCMD-3 DSM-IV | 30/30 | 22/38 | A: 49 (15) C: 48 (15) | A: 3.6 (2.4) years C: 3.9 (2.4) years | Manual | 8 weeks | Sham A | HAMD; HAMA; PAC-SYM; Clinical Efficacy | 4 weeks | 2/2 | NI |
| 28^28^ | Wang, et al. | 2013 | Depression | DSM-IV | 24/24 | 15/33 | A: 48.1 (13.4) C: 47.1 (10.6) | A: 9.5 (9.2) months C: 15.8 (21.3) months | Electro | 24 weeks | Medication | MMPI; MADRS; SDS | 6 weeks | 6/6 | Y |
| 29^29^ | Fu, et al. | 2009 | Depression | CCMD | 176/176/88 | 149/291 | A: 41.9 (12.3) C: 39.9 (11.0) C: 43.5 (11.4) | A: 4.6 (3.0) years C: 3.9 (2.4) years C: 4.6 (2.8) years | Manual | 3 months | Medication Sham A | SDS; SERS | NA | 0/0 | N |
| 30^30^ | Wong, et al. | 2021 | Depression | DSM-V | 47/20 | 21/49 | A: 50.9 (11.1) C: 44.8 (10.3) | A: 12.4 (12.1) years C: 10.3 (8.5) years | Manual | 3 weeks | Waitlist | HAMD-17; PHQ-9 | NA | 3/0 | N |
| 31^31^ | Zhao, et al. | 2020 | Depression Insomnia | DSM-V | 34/33 | 24/43 | A: 43.9 (10.5) C: 45.2 (11.4) | A: 4.7 (1.4) months C: 4.0 (1.5) months | Manual | 8 weeks | Sham Acupuncture | PSQI; HAMD-17 | NA | 0/0 | N |
| 32^32^ | Yan, et al. | 2023 | Depression Insomnia | DSM-V ICD-10 | 30/30 | 18/39 | A: 37 (12) C: 34 (10) | A: 15.3 (5.4) months C: 14.8 (6.6) months | Manual | 4 weeks | rTMS plus Sham A | HAMD-17; PSQI | 1 month | 1/2 | N |
| 33^33^ | Yan, et al. | 2023 | Depression Insomnia | DSM-V | 30/30 | 16/44 | A: 36 (11) C: 33 (10) | A: 12.8 (6.8) months C: 13.0 (6.4) months | Manual | 8 weeks | Sham A | SDS; ISI | 1 month | 0/0 | N |
| 34^34^ | Gao, et al. | 2023 | Treatment resistant depression | DSM-V | 39/39 | 32/39 | A: 42 (12) C: 38 (11) | A: 13.3 (8.6) months C: 14.7 (7.6) months | Manual | 8 weeks | Medication | HAMD-24; HAMA; VAS | 12 weeks | 3/4 | NI |
| 35^35^ | Zhao, et al. | 2014 | Anxiety | CCMD-3-R | 30/30 | 24/36 | A: 42.3 (8.4) C: 41.6 (7.9) | A: 14.3 (5.6) months C: 16.6 (5.3) months | Manual | 6 weeks | Medication | HAMA; SAS; Clinical Efficacy; TESS | NA | 0/0 | Y |
| 36^36^ | Chen, et al. | 2013 | Anxiety | CCMD-3-R | 32/31 | 21/45 | A: 45.3 (11.0) C: 45.1 (10.7) | A: 13.4 (12.0) months C: 14.0 (11.8) months | Electro | 6 weeks | Medication | HAMA; Clinical Efficacy; SERS | NA | 1/2 | Y (SERS) |
| 37^37^ | Che, et al. | 2015 | Anxiety | CCMD-3 | 40/40 | 32/48 | O: 43.2 (7.8) | O: 18.4 (10.1) months | Manual | 6 weeks | Medication | HAMA; SAS; TESS | NA | 0/0 | Y |
| 38^38^ | Wang | 2007 | Anxiety | CCMD-3 | 21/20 | 17/24 | NI | NI | Manual | 30 days | Medication | HAMA; Clinical Efficacy | NA | 0/0 | NI |
| 39^39^ | Zhou, et al. | 2013 | Anxiety | CCMD-3 | 40/40 | 34/46 | A: 38 (8) C: 38 (10) | A: 8.7 (2.2) months C: 8.6 (3.0) months | Manual | 6 weeks | Medication | HAMA; Clinical Efficacy | NA | 0/0 | NI |
| 40^40^ | Zheng, et al. | 2004 | Anxiety | CCMD-2 | 58/50 | 28/80 | A: 35 (5.2) C: 34 (6.1) | A: 12 (5.3) months C: 11 (6.8) months | Manual | 8 weeks | Medication | HAMA; Clinical Efficacy | NA | 0/0 | Y |
| 41^41^ | Zhao, et al. | 2018 | Anxiety | CCMD-3 | 55/59 | 43/71 | A: 42.6 (8.2) C: 43.3 (8.0) | A: 16.5 (4.8) months C: 15.4 (4.5) months | Electro | 6 weeks | Medication | HAMA; Clinical Efficacy; TESS | NA | 5/1 | Y |
| 42^42^ | Liu, et al. | 2020 | Anxiety | CCMD-3 | 50/50/50/50 | 85/115 | A: 38 (3) C: 37 (3) C: 38 (2) C: 38 (3) | A: 14.0 (2.4) months C: 13.0 (3.1) months C: 13.5 (2.7) months C: 13.6 (2.6) months | Manual | 60 days | C1: CES C2: CES plus A C3: Medication | HAMA; WHOQOL-BREF; TESS | 1 year | 0/0 | Y (TESS) |
| 43^43^ | Liu, et al. | 2007 | Anxiety | CCMD-3-R | 27/26/26 | 30/56 | O: 38.5 (15.8) | O: 10.3 (7.5) months | Manual | 6 weeks | C1: Medication C2: Medication plus A | HAMA; SAS; CGI; TESS | NA | 2/3/2 | Y |
| 44^44^ | Tan, et al. | 2017 | Mild cognitive impairment | DSM-IV | 16/16 | 16/16 | A: 65.9 (4.7) C: 64.6 (5.3) | NI | Manual | 4 weeks | Sham A | MMSE; MoCA; ADAS-Cog; digit-symbol substitution test, digit-span test | NA | 0/0 | NI |
| 45^45^ | Chen, et al. | 2019 | Mild cognitive impairment | DSM-IV ICD-10 | 32/32/32 | 46/50 | A: 71 (5) C: 71 (5) C: 71 (5) | A: 70.4 (80.0) months C: 76.6 (58.5) months C: 74.0 (92.5) months | Manual | 8 weeks | C1: Sham A C2: Waitlist | MMSE; MoCA | 2 months | 0/0 | NI |
| 46^46^ | Zhao, et al. | 2012 | Mild cognitive impairment | DSM-IV ICD-10 | 94/93 | 90/97 | A: 69 (7) C: 67 (6) | NI | Electro | 8 weeks | Medication | MMSE; GRT; Clinical efficacy | 6 mouths | 2/3 | Y |
| 47^47^ | Zhao, et al. | 2010 | Mild cognitive impairment | DSM-IV ICD-10 | 30/31 | 29/32 | A: 67.4 (6.6) C: 69.3 (7.3) | NI | Electro | 8 weeks | Medication | MMSE; Clinical efficacy | NA | 2/1 | Y |
| 48^48^ | Zhang, et al. | 2013 | Mild cognitive impairment | Clinical symptoms | 78/75 | 70/83 | A: 71 (8) C: 72 (9) | NI | Electro | 8 weeks | Medication | MMSE; Picture recognition; CDT | NA | 0/0 | NI |
| 49^49^ | Liu, et al. | 2010 | Mild cognitive impairment | DSM-IV | 17/19 | 16/20 | A: 66.0 (6.8) C: 69.3 (6.9) | NI | Electro | 30 days | Medication | MMSE; CDT; ADL; GDS; CDR | NA | O: 14 | NI |
| 50^50^ | Li, et al. | 2023 | Mild cognitive impairment | DSM-III-R NINCDS-ADRDA | 30/30/30 | 35/55 | A: 64.5 (7.4) C: 62.8 (7.7) A+C: 63.3 (6.4) | A: 48.4 (21.7) months C: 47.9 (18.3) months A+C: 46.1 (17.1) months | Manual | 4 weeks | C1: Medication C2: Medication plus A | MMSE; MoCA | NA | 0/0/0 | Y |
| 51^51^ | Karst, et al. | 2001 | Tension-type headache | IHS | 34/35 | 31/38 | A: 47.9 (13.8)  C: 48.2 (14.6) | NI | Manual | 5 weeks | Sham A | VAS; CGI; ELQ; LS; DS; NHP; FQCI; Clinical efficacy | 5 months | 0/0 | NI |
| 52^52^ | Wang, et al. | 2017 | Migraine | ICHD-3 | 19/19 | 8/30 | A: 30 (6) C: 31 (7) | A: 7.0 (3.2) years C: 7.4 (3.5) years | Manual | 4 weeks | Sham A | VAS; Clinical efficacy | 4 weeks | 1/1 | NI |
| 53^53^ | Zhang, et al. | 2019 | Migraine | ICHD-3β | 18/17 | 5/30 | A: 39.7 (12.3) C: 33.2 (9.5) | A: 13.0 (0.6) years C: 13.2 (0.6) years | Manual | 4 weeks | Sham A | Heat pain threshold; Clinical efficacy | NA | 0/0 | NI |
| 54^54^ | Wang, et al. | 2015 | Migraine | IHS | 26/24 | 13/37 | A: 41.6 (14.9) C: 43.8 (13.4) | A: 18.4 (12.7) years C: 21.1 (13.3) years | Manual | 20 weeks | Sham A | VAS; Likert scale; McGill; MSQOL; Clinical efficacy | 1 year | 1/1 | N |
| 55^55^ | Streng, et al. | 2006 | Migraine | IHS | 59/54 | 13/100 | A: 40.0 (11.4) C: 40.3 (10.7) | A: 14.5 (9.9) years C: 17.3 (10.8) years | Manual | 12 weeks | Medication | PDI; SES; ADS; SF- 36; Clinical efficacy | 12 weeks | 2/17 | Y |
| 56^56^ | Giannini, et al. | 2021 | Migraine | IHS | 69/66 | 21/114 | A: 33.6 (17.4) C: 34.7 (16.5) | A: 23.9 (10.0) years C: 24.3 (9.8) years | Manual | 4 months | Medication | Zung scales; MIDAS; SF-36; Clinical efficacy | 6 months | 12/20 | Y |
| 57^57^ | Musil, et al. | 2018 | Migraine | IHS | 42/44 | 10/76 | A: 45.6 (12.8) C: 46.5 (10.3) | A: 26.9 (12.9) years C: 23.0 (14.1) years | Manual | 12 weeks | Waitlist | VAS; MIDAS; Clinical efficacy | 6 months | 4/1 | Y |
| 58^58^ | Melchart, et al. | 2005 | Tension-type headache | IHS | 132/63/75 | 71/199 | A: 42.3 (13.5) C: 43.4 (12.9) C: 42.8 (13.2) | A: 13.7 (11.1) years C: 16.8 (13.8) years C: 14.1 (11.1) years | Manual | 8 weeks | C1: Sham A C2: Waitlist | SES; ADS; SF-36; Clinical efficacy | 12 weeks | 8/4/10 | Y |
| 59^59^ | Linde, et al. | 2005 | Migraine | IHS | 145/81/76 | 35/267 | A: 43.3 (11.8) C: 41.3 (10.2) C: 42.5 (11.8) | A: 20.9 (12.1) years C: 19.2 (11.7) years C: 19.7 (11.3) years | Manual | 8 weeks | C1: Sham A C2: Waitlist | PDI; SES; ADS; SF-36; Clinical efficacy | 24 weeks | 7/3/10 | Y |
| 60^60^ | Sozen, et al. | 2013 | Chronic headache | ICHD-II | 34/22 | 4/52 | A: 43.7 (8.9) C: 41.3 (9.8) | A: 14.4 (7.1) years C: 12.5 (11.2) years | Manual | 3 months | Sham A | VAS; Clinical efficacy | NA | 0/0 | Y |
| 61^61^ | Cai, et al. | 2022 | Headache | ICHD-3 | 53/53 | 17/89 | A: 42 (9) C: 41 (9) | A: 10.8 (4.4) years C: 11.4 (5.3) years | Electro | 4 weeks | Medication | VAS; Clinical efficacy | 6 months | 2/2 | Y |
| 62^62^ | Ferro, et al. | 2012 | Chronic migraine | IHS | 22/23/23 | 0/68 | A: 38.2 (7.4) C: 37.3 (8.6) A+C: 40.6 (9.1) | A: 12.2 (3.8) years C: 11.6 (5.6) years A+C: 12.4 (3.2) years | Manual | 10 weeks | C1: Medication C2: Medication plus A | VAS; MIDAS; SF-36 | NA | 1/0/0 | N |
| 63^63^ | Xu, et al. | 2020 | Headache | ICHD-3β | 58/60/29 | 27/123 | A: 36.6 (12.0) C: 36.0 (10.9) C: 37.3 (11.7) | Median (IQR) A: 10.0 (5.0-19.5) years C: 10.0 (6.0-14.0) years C: 16.5 (7.8-23.0) years | Manual | 8 weeks | C1: Sham A C2: Usual care | MSQOL; PSQI; MIDAS; BAI; BDI-II; Clinical efficacy | 4 weeks | 3/1/2 | Y |
| 64^64^ | Zheng, et al. | 2022 | Chronic tension-type headache | IHS | 110/108 | 61/157 | A: 43.0 (12.5) C: 43.2 (12.8) | A: 131 (131) months C: 129 (121) months | Manual | 8 weeks | Sham A | VAS; Clinical efficacy | 32 weeks | 0/0 | Y |
| 65^65^ | Liu, et al. | 2022 | Migraine | ICHD-3β | 21/21 | 4/36 | A: 38.60 (12.27) C: 36.10 (9.61) | A: 16.30 (10.79) years C: 14.50 (7.04) years | Manual | 4 weeks | Sham A | MMDs; VAS; HIT-6; HRQOL; MSQOL; BDI-II; BAI; PSQI; MoCA; Clinical efficacy | 4 weeks | 1/1 | NI |
| 66^66^ | Feng, et al. | 2023 | Migraine | ICHD-3 | 38/38 | 25/39 | A: 42.73 (8.92) C: 43.82 (9.63) | A: 3.82 (1.33) years C: 3.41 (0.91) years | Manual | 4 weeks | Medication | VAS; Clinical efficacy | NA | 0/0 | N |
| 67^67^ | Karst, et al. | 2000 | Chronic tension-type headache | IHS | 21/18 | 20/19 | A: 50.4 (13.5) C: 47.3 (16.5) | NI | Manual | 5 weeks | Sham A | VAS; CGI; ELQ; DS; NHP; FQCI; Clinical efficacy | 6 weeks | 0/0 | NI |
| 68^68^ | Allais, et al. | 2003 | Chronic headache | Clinical symptoms | 18/18 | 0/36 | O: 41.4 (10.3) | NI | Manual | 8 weeks | TENS | Clinical efficacy | NA | 2/2 | NI |
| 69^69^ | Zhao, et al. | 2018 | Insomnia | DSM-V | 34/32 | 17/49 | A: 51.67 (8.70) C: 50.96 (8.46) | NI | Electro | 5 weeks | Sham A | PSQI; PSG | 4 weeks | 2/4 | NI |
| 70^70^ | Wang, et al. | 2021 | Insomnia | ICSD-3 | 30/29 | 24/35 | A: 69 (4) C: 69 (5) | A: 19.9 (6.0) years C: 19.7 (5.7) years | Electro | 4 weeks | Sham A | PSQI; MoCA | NA | 0/1 | NI |
| 71^71^ | Wang, et al. | 2020 | Insomnia | Clinical symptoms | 43/43 | 16/70 | A: 51.5 (12.1) C: 51.1 (11.6) | NI | Electro | 5 weeks | Sham A | PSQI; AIS; PSG | NA | 0/0 | NI |
| 72^72^ | Xi, et al. | 2021 | Chronic insomnia | ICSD-3 | 29/29 | 25/33 | A: 44 (12) C: 41 (12) | A: 6.6 (4.4) years C: 5.7 (4.7) years | Electro | 4 weeks | Sham A | PSQI; PSAS; HAS | NA | 1/1 | Y |
| 73^73^ | Xiao, et al. | 2013 | Insomnia | DSM-IV | 20/20 | 19/21 | A: 39.6 (11.3) C: 40.8 (10.8) | A: 38.9 (20.8) months C: 40.0 (20.8) months | Manual | 14 days | Sham A | PSQI; Clinical Efficacy | NA | 0/0 | Y |
| 74^74^ | Yeung, et al. | 2009 | Insomnia | DSM-IV | 30/30 | 14/46 | A: 48.3 (9.5) C: 47.8 (8.6) | A: 7.7 (8.1) years C: 10.8 (16.7) years | Electro | 3 weeks | Sham A | ISI; PSQI; SOL; TST; WASO; HADS; SE; Sleep quality | NA | 1/2 | Y |
| 75^75^ | Yin, et al. | 2020 | Depression-related insomnia | DSM-IV | 30/30 | 21/39 | A: 47.3 (14.9) C: 49.8 (15.1) | A: 5.7 (5.7) years C: 7.5 (6.2) years | Electro | 8 weeks | Sham A | PSQI; HAMD-17; SDS; HAMA; SE; TST; SA | 4 weeks | 3/4 | Y |
| 76^76^ | Zhang, et al. | 2020 | Insomnia | DSM-IV | 46/44 | 43/53 | A: 36.6 (14.4) C: 39.2 (13.8) | A: 20.6 (18.9) months C: 21.6 (16.9) months | Manual | 2 weeks | Sham A | PSQI; VAS; SAS; SDS; Clinical efficacy | 1 month | 2/4 | Y |
| 77^77^ | Zhao, et al. | 2021 | Chronic insomnia | ICSD-3 | 29/27 | 23/33 | A: 47.2 (14.1) C: 45.6 (12.7) | A: 2.7 (3.2) years C: 2.9 (3.6) years | Electro | 4 weeks | Sham A | PSQI; HAS | 1 month | 1/3 | NI |
| 78^78^ | Chen, et al. | 2015 | Insomnia | CDC-CFS | 43/24 | 27/40 | A: 34.9 (9.2) C: 33.1 (9.3) | A: 30.6 (1.8) months C: 34.9 (64.1) months | Manual | 4 weeks | Sham A | PSQI; Morningness Questionaire | NA | 4/1 | NI |
| 79^79^ | Luo, et al. | 2017 | Insomnia Depression | DSM-V | 36/34 | 17/53 | A: 48.6 (12.5) C: 49.5 (9.2) | (Insomnia) A: 83.1 (66.0) months C: 76.6 (63.1) months (Depression) A: 16.0 (66.0) months C: 23.2 (17.7) months | Manual | 8 weeks | Sham A | PSQI; HAMD; Clinical efficacy | 4 weeks | 4/6 | Y |
| 80^80^ | Zhang, et al. | 2018 | Insomnia Depression | CCMD-3 | 33/34 | 32/35 | A: 31.4 (13.2) C: 35.6 (10.3) | NI | Manual | 10 days | Sham A | PSQI; SDS; Clinical efficacy | 3 month | 1/1 | Y |
| 81^81^ | Dong, et al. | 2018 | Insomnia | ICD-10 ICSD-2 DSM-IV | 32/31 | 35/37 | A: 45 (18) C: 44 (21) | A: 1.6 (1.4) years C: 1.7 (1.3) years | Manual | 4 weeks | Sham A | PSQI; Clinical Efficacy | 4 weeks | 4/5 | NI |
| 82^82^ | Zhao, et al. | 2018 | Chronic insomnia | ICSD-3 DSM-V CCMD-3 | 30/30 | 26/34 | A: 36.8 (10.7) C: 38.4 (10.8) | A: 4.6 (1.2) months C: 4.7 (1.3) months | Manual | 8 weeks | Sham A | PSQI; ERPs | NA | 0/0 | Y |
| 83^83^ | Li, et al. | 2009 | Insomnia | CCMD-2-R | 30/30 | 21/39 | A: 30.1 (2.3) C: 32.2 (1.8) | A: 19 (5.3) months C: 18 (5.1) months | Manual | 2 weeks | Sham A | PSQI | 1 month | 2/3 | Y |
| 84^84^ | Yin, et al. | 2017 | Insomnia | CCMD-3 | 30/30 | 24/36 | A: 43.4 (13.5) C: 46.8 (13.0) | A: 19.9 (7.2) months C: 19.9 (6.7) months | Manual | 4 weeks | Medication | PSQI; Clinical Efficacy | NA | 0/0 | NI |
| 85^85^ | Chen, et al. | 2013 | Insomnia | CCMD-3 | 59/57 | 48/68 | A: 45.6 (9.5) C: 42.9 (9.4) | A: 11.6 (7.2) months C: 10.7 (7.1) months | Manual | 4 weeks | Medication | PSQI | 1 month | 1/3 | Y |
| 86^86^ | Yang, et al. | 2011 | Insomnia | CCMD-3 | 37/31 | 28/40 | A: 50.0 (0.7) C: 51.0 (0.2) | NI | Manual | 30 days | Medication | PSQI; Clinical Efficacy | 12 months | 0/0 | NI |
| 87^87^ | Zhou, et al. | 2016 | Insomnia | Clinical symptoms | 34/34 | 39/29 | A: 52.2 (5.7) C: 52.5 (5.8) | A: 26.1 (6.8) months C: 25.3 (7.5) months | Manual | 28 days | Medication | PSQI | NA | 0/0 | NI |
| 88^88^ | Liu, et al. | 2017 | Insomnia | DSM-V | 31/30 | 20/41 | A: 47 (10) C: 47 (10) | A: 14.0 (6.5) months C: 14.0 (6.3) months | Manual | 4 weeks | Medication | PSQI; PSG; MSLT | NA | 1/2 | NI |
| 89^89^ | Pan, et al. | 2017 | Insomnia | ICD-10 ICSD-2 DSM-IV | 40/40 | 47/33 | A: 55.5 (3.1) C: 55.7 (3.2) | A: 1 (0.4) years C: 1 (0.2) years | Manual | 14 days | Medication | PSQI; Clinical Efficacy | NA | 0/0 | NI |
| 90^90^ | Xu, et al. | 2020 | Insomnia | CCMD-3 | 30/30 | 23/37 | A: 48.3 (7.8) C: 47.4 (8.1) | A: 30.4 (18.2) days C: 32.3 (19.2) days | Manual | 4 weeks | Medication | PSQI; Clinical Efficacy | NA | 0/0 | NI |
| 91^91^ | Liu, et al. | 2016 | Insomnia | ICD-10 CCMD-3 | 32/31 | NI | A: 43 (6) C: 44 (5) | A: 14 (5.2) months C: 14 (5.5) months | Manual | 20 days | Medication | PSQI; Clinical Efficacy | NA | 2/3 | Y |
| 92^92^ | Liu, et al. | 2015 | Insomnia | ICD-10 CCMD-3 | 96/95 | NI | NI | NI | Manual | 20 days | Medication | PSQI; Clinical Efficacy | NA | 2/3 | Y |
| 93^93^ | Tu, et al. | 2012 | Insomnia | DSM-IV | 19/14 | 15/18 | A: 53.68 (16.7) C: 53.43 (17.0) | NI | Manual | 4 weeks | Medication | PSQI | NA | 2/2 | N |
| 94^94^ | Wu, et al. | 2021 | Insomnia | ICSD-3 | 30/29 | 22/37 | A: 41 (10) C: 42 (10) | A: 13.4 (5.3) months C: 14.2 (4.8) months | Electro | 4 weeks | Medication | PSQI; Clinical Efficacy | NA | 0/1 | NI |
| 95^95^ | Ji, et al. | 2015 | insomnia | ICD-10 | 35/35 | 36/34 | A: 37 (11) C: 36 (13) | A: 1.8 (1.2) years C: 1.7 (1.4) years | Manual | 4 weeks | Medication | PSQI; Clinical Efficacy; SERS | NA | 0/0 | NI |
| 96^96^ | Guo, et al. | 2008 | Insomnia | ICD-10 | 30/30 | 26/34 | A: 49.1 (11.6) C: 52.1 (9.3) | A: 57.7 (51.1) months C: 53.2 (26.4) months | Manual | 30 days | Medication | Epworth; PSQI | NA | 0/0 | NI |
| 97^97^ | He, et al. | 2010 | Insomnia | ICD-10 CCMD-2 | 30/30 | 18/42 | A: 35.6 (10.6) C: 33.5 (9.4) | A: 10.9 (7.6) months C: 9.6 (7.6) months | Manual | 4 weeks | Medication | PSQI; SDS; SAS | NA | 0/0 | NI |
| 98^98^ | Zhao, et al. | 2017 | Insomnia | Clinical symptoms | 58/40 | 52/46 | A: 40.8 (12.2) C: 40.0 (10.5) | A: 13 (12.8) months C: 13.6 (11.5) months | Manual | 30 days | Medication | PSQI; Clinical Efficacy | NA | 0/0 | NI |
| 99^99^ | Wang, et al. | 2019 | Refractory insomnia | ICD-10 ICSD-2 DSM-IV | 60/60 | 58/62 | A: 42 (11) C: 41 (11) | A: 48.2 (20.7) months C: 45.6 (19.4) months | Manual | 28 days | Medication | PSQI; Clinical Efficacy | NA | 0/0 | NI |
| 100^100^ | Wang, et al. | 2015 | Insomnia | CCMD-3 | 33/30/35 | 41/57 | A: 73 (6) C: 73 (6) A+C: 73 (6) | A: 15.0 (7.1) months C: 15.1 (7.3) months A+C: 15.0 (6.9) months | Manual | 4 weeks | C1: Medication C2: Medication plus A | PSQI | 4 weeks | 0/0 | Y |
| 101^101^ | Zhang, et al. | 2019 | Insomnia | ICSD-2 | 90/90/90 | NI | A: 72 (9) C: 72 (10) A+C: NI | NI | Manual | 21 days | C1: Medication C2: Medication plus A | PSQI; PSG; Clinical Efficacy | 15 days | 11/10/7 | NI |
| 102^102^ | Luo, et al. | 2010 | Insomnia Depression | CCMD-3 | 33/32 | 21/44 | A: 51.3 (14.4) C: 50.0 (17.3) | A: 10.1 (9.9) months C: 11.0 (10.1) months | Manual | 4 weeks | Medication | PSQI; SDS; Clinical Efficacy; SERS | NA | 2/3 | Y |
| 103^103^ | Zhang, et al. | 2020 | Depression Insomnia | CCMD-3 | 30/30 | NI | NI | NI | Manual | 10 days | Sham A | PSQI; SDS | 1 month | 0/1 | Y |
| 104^104^ | Li | 2016 | Insomnia | CCMD-3 | 35/35 | 29/41 | A: 44 (4) C: 42 (3) | A: 17.3 (5.2) months C: 16.4 (4.3) months | Manual | 2 weeks | Acu-pressure | PSQI; Clinical Efficacy | 2 weeks | 0/0 | NI |
| 105^105^ | Yin, et al. | 2017 | Insomnia | DSM-IV | 36/36 | 32/40 | A: 39.7 (12.9) C: 37.3 (15.1) | NI | Manual | 4 weeks | Sham A | ISI; SE; SA; TST; SAS; SDS | 4 weeks | 4/6 | Y |
| 106^106^ | Feng, et al. | 2020 | Chronic insomnia | ICSD-3 | 45/45/45 | 46/89 | A: 46 (11) C: 47 (10) C: 47 (10) | A: 13.9 (6.0) months C: 14.3 (6.2) months C: 14.1 (6.1) months | Manual | 4 weeks | C1: Medication C2: Sham A | ISI; AVMT; PSG | NA | 1/2/2 | Y |
| 107^107^ | Wu, et al. | 2014 | Chronic insomnia | ICD-10 | 20/20 | 5/35 | A: 50.0 (14.3) C: 50.6 (15.4) | A: 14.9 (8.5) months C: 16.9 (9.8) months | Manual | 4 weeks | Medication | ESS; Clinical Efficacy | NA | 0/0 | N |
| 108^108^ | Huo, et al. | 2023 | Insomnia | DSM-IV | 30/30 | 30/30 | A: 48 (9) C: 46 (8) | Median (IQR) A: 39 (21-51) months C: 36 (12-48) months | Manual | 4 weeks | Sham A | MoCA; DST; TMT; PSQI; FS-14 | 4 weeks | 2/3 | NI |
| 109^109^ | Zhang, et al. | 2023 | Insomnia | DSM-V | 44/46 | 32/58 | A: 38.09 (13.33) C: 39.41 (13.93) | NI | Manual | 2 weeks | Sham A | PSQI; SAS; SDS; Clinical Efficacy | 6 months | 1/1 | NI |
| 110^110^ | Chen, et al. | 2023 | Insomnia | DSM-V | 26/24 | 18/32 | A: 31.50 (18) C: 40.00 (20) | NI | Manual | 4 weeks | Sham A | PSQI; HAS; TST; WASO; Clinical Efficacy | NA | O: 10 | NI |

^a^Data in mean (SD)

Abbreviations: A, Acupuncture; AC, Acupuncture; ADAS-Cog, Alzheimer’s Disease Assessment Scale-Cognitive Subscale; ADL, Activities of Daily Living Scale; ADS, Allgemeine Depressions-Skala; AIS, Athens Insomnia Scale; AVMT, Auditory Verbal Memory Test; BAI, Beck Anxiety Inventory; BDI-II, Beck Depression Inventory II; C, Control; CCMD, Chinese Classification of Mental Disorders; CDC, Centers for Disease Control and Prevention; CDR, Clinical Dementia Rating Scale; CDT, Clock Drawing Test; CES, Cranial Electrotherapy Stimulation; CFS, Chronic Fatigue Syndrome; CGI, Clinical Global Impressions; CGIS, Clinical Global Impression Scale; DS, von Zerssen Depression Scale; DSI, Depressive Severity Index; DSM: Diagnostic and Statistical Manual; ELQ, Everyday-Life-Questionnaire; ERPs, Event Related Potentials; ESS, Epworth Sleepiness Scale; FAI, Fatigue Assessment Index; FQCI, Freiburg Questionnaire of Coping with Illness; FS, Fatigue Scale; FS-14, Fatigue Scale 14; FSS, Fatigue Severity Scale; GDS, Global Deterioration Scale; GHQ-12, General Health Questionnaire 12; GRT, Graphic Recognition Test; HADS, Hospital Anxiety and Depression Scale; HAMA, Hamilton Anxiety Scale; HAMD, Hamilton Depression Scale; HAS, Hyperarousal Scale; HIS, International Headache Society; HIT-6, Headache Impact Test-6; HRQOL, Health-Related Quality of Life; ICD, International Classification of Diseases; ICHD, International Classification of Headache Disorders; ICSD, International Classification of Sleep Disorders; HIS, International Headache Society; ISCD-3, International Classification of Sleep Disorders, 3rd edition; ISI, Insomnia Severity Index; LS, Life Quality Scale; MADRS, Montgomery-Asberg Depression Rating Scale; MIDAS, Migraine Disability Assessment Score; MMDs, Monthly Migraine Days, MMPI, Minnesota Multiple Personality Inventory; MMSE, Mini-Mental State Examination; MoCA, Montreal Cognitive Assessment; MSLT, Multiple Sleep Latency Test; MSQOL, Migraine Specific Quality of Life; NHP, Nottingham Health Profile; NI, No Information; NINCDS-ADRDA, National Institute of Neurological and Communicative Disorders and Stroke and the Alzheimer's Disease and Related Disorders Association; O, Overall; PAC-SYM, Patient Assessment of Constipation-Symptoms; PDI, Pain Disability Index; PHQ-9, Patients Health Questionnaire; PSAS, Pre-Sleep Arousal Scale; PSG, Polysomnography; PSQI, Pittsburgh Sleep Quality Index; SA, Sleep Awakenings; SAS, Self-rating Anxiety Scale; SCL-90, Symptom Check-List 90; SDS, Self-rating Depression Scale; SE, Sleep Efficiency; SERS, Side Effect Rating Scale; SES, Schmerzempfindungs-Skala; SF-12, Short Form 12; SOL, Sleep-Onset Latency; SPHERE, Somatic and Psychological Health Report; TENS, Transcutaneous Electrical Nerve Stimulation; TESS, Treatment Emergent Symptom Scale; TST, Total Sleep Time; VAS, Visual Analogue Scale; WASO, Wake Time After Sleep Onset; WHOQOL-BREF, World Health Organization's Quality of Life Questionnaire, Brief version.

**References:**

1. Wang JJ, Song YJ, Wu ZC, et al. Randomized controlled clinical trials of acupuncture treatment of chronic fatigue syndrome. *Zhen Ci Yan Jiu*. 2009;34(2):120-124.

2. Chen S san, Liu R, Wu B, et al. Acupuncture on back-shu points of five zang for chronic fatigue syndrome: A randomized control trial. *World Journal of Acupuncture - Moxibustion*. 2018;28(4):237-241. doi:10.1016/j.wjam.2018.12.007

3. Zheng SH, Zheng SZ, Jiao JK, et al. Randomized controlled clinical trial of back-shu point and front-mu point acupuncture for treatment of chronic fatigue syndrome. *Liaoning Journal of Traditional Chinese Medicine*. 2012;39(4):726-728.

4. An GX. Clinical analysis of back-shu point and front-mu point acupuncture for treatment of chronic fatigue syndrome. *Chinese Journal of Basic Medicine in Traditional Chinese Medicine*. 2014;20(7):970-971+991.

5. Wu WZ, Zhao YN, Liu CY, et al. Effects of electroacupuncture on sleep quality, daytime fatigue and serum cortisol in chronic insomnia. *CJTCMP*. 2021;36(9):5693-5696.

6. Ma J, Yue S, Wang XX, Fan D, Chen HL, Zhang L. Clinical study of smokeless moxibustion for treatment of chronic fatigue syndrome. *Journal of Clinical Medical*. 2018;5(28):106-107+109.

7. Tian L, Wang J, Luo C, et al. Moxibustion at Gaohuang (BL 43) for chronic fatigue syndrome: a randomized controlled trial. *Zhongguo Zhen Jiu*. 2015;35(11):1127-1130.

8. Song YJ, Wang JJ, Wang QM, Wang XH, Wu ZC, Meng H. Randomized controlled study on influence of acupuncture for pain degree of patients with chronic fatigue syndrome. *Chinese Journal of Information on TCM*. 2010;17(10):6-8.

9. Zhu YH, Liang FR, Cheng CS, Wu X, Wang HW, Bao KY. A randomized controlled trial of electroacupuncture for chronic fatigue syndrome. *SHJTCM*. 2008;42(10):48-50. doi:10.16305/j.1007-1334.2008.10.018

10. Jiang S, Fan X. Study of acupuncture on chronic fatigue syndrome. *Trauma and Critical Care Medicine*. 2017;5(6):350-353. doi:10.16048/j.issn.2095-5561.2017.06.09

11. Li ZX, Zhang Y, Yan L da, et al. Effect of electroacupuncture at back-shu points of five zang on fatigue status and cortical excitability in chronic fatigue syndrome. *Zhongguo Zhen Jiu*. 2022;42(11):1205-1210. doi:10.13703/j.0255-2930.20220124-k0006

12. Li SJ, Liu T. Clinical observation on treatment of melancholia by acupuncture following principle of relieving depression and regulating mentality. *Zhongguo Zhong Xi Yi Jie He Za Zhi*. 2007;27(2):155-157.

13. Ma Q, Zhou DA, Wang LP. Clinical curative effect and factor analysis of depression treated by acupuncture. *Zhongguo Zhen Jiu*. 2011;31(10):875-878.

14. Wei L, Zheng ZY, Liu D, Chen HL, Jin H, Yang JF. RCTs of Fuyang Yiyin Acupuncture in Treating Depression. *JCAM*. 2021;37(8):30-34. doi:10.19917/j.cnki.1005-0779.021157

15 Yang Y, Zhang J. Acupuncture at five-zang back-shu points and Geshu for depression: a randomized controlled trial. *Beijing Journal of Traditional Chinese Medicine*. 2015;34(1):14-17. doi:10.16025/j.1674-1307.2015.01.004

16. Xie YC, Li YH. Observation on therapeutic effect of acupuncture at Zhongwan (CV 12) and Si-guan points combined with reinforcing-reducing manipulation of respiration for treatment of depression. *Zhongguo Zhen Jiu*. 2009;29(7):521-524.

17. Yan M, Mao X, Wu JL. Comparison of electroacupuncture and amitriptyline in treating depression. *Chinese Journal of Clinical Rehabilitation*. 2004;8(18):3548-3549.

18. Zhang LJ, Zhao H. Clinical observation of acupunture in treating depression and effect on serum cell factors. *Chinese Journal of Information on Traditional Chinese Medicine*. 2007;14(6):15-17.

19. Zhu PY, Xue H. Clinical observation on the efficacy of acupuncture for relieving the depressed liver and regulating qi in the treatment of depression among seafarers. *Chin J Naut Med & Hyperbar Med*. 2016;23(5):360-361. doi:10.3760/cma.j.issn.1009-6906.2016.05.008

20. Duan DM, Tu Y, Jiao S, Qin W. The relevance between symptoms and magnetic resonance imaging analysis of the hippocampus of depressed patients given electro-acupuncture combined with Fluoxetine intervention - A randomized, controlled trial. *Chin J Integr Med*. 2011;17(3):190-199. doi:10.1007/s11655-011-0666-6

21. Luo RH, Xu K, Huang YS. Clinical Observations on Acupuncture Treatment for Depression. *Shanghai Journal of Acupuncture and Moxibustion*. 2009;28(2):69-71. doi:10.13460/j.issn.1005-0957.2009.02.003

22. Du Y hao, Li G ping, Yan H, Zhang X jun, Huang L fang. Clinical study on needling method for regulating mental activities and soothing liver for treatment of melancholia. *Zhongguo Zhen Jiu*. 2005;25(3):151-154.

23. Yi Y, Xu F ming, Xie H wu. Correlation between the liver meridian and the frontal lobe in depression by needling at taichong (LV3): a resting-state fMRI study. *Zhongguo Zhong Xi Yi Jie He Za Zhi*. 2011;31(8):1044-1050.

24. Duan D mei, Tu Y, Chen L ping. Assessment of effectiveness of electroacupuncture and fluoxetine for treatment of depression with physical symptoms. *Zhongguo Zhen Jiu*. 2008;28(3):167-170.

25. Liu C, Zhao Y, Qin S, Wang X, Jiang Y, Wu W. Randomized controlled trial of acupuncture for anxiety and depression in patients with chronic insomnia. *Ann Transl Med*. 2021;9(18):1426. doi:10.21037/atm-21-3845

26. Ma J, Tao SP, Chu XY, et al. Efficacy and safety of acupuncture treatment on depression by unblocking du meridian and relieving depression: a randomized controlled trial. *Liaoning Journal of Traditional Chinese Medicine*. 2020;47(5):180-182. doi:10.13192/j.issn.1000-1719.2020.05.054

27. Yin P, Ma J, Wu HG, Xu SF. Clinical observation of Jie Yu Tong Fu needling for depression coupled with constipation. *Shanghai Journal of Acupuncture and Moxibustion*. 2018;37(2):159-164. doi:10.13460/j.issn.1005-0957.2018.02.0159

28. Wang W dong, Lu X yu, Ng S man, et al. Effects of electro-acupuncture on personality traits in depression: A randomized controlled study. *Chin J Integr Med*. 2013;19(10):777-782. doi:10.1007/s11655-013-1594-4

29. Fu W bin, Fan L, Zhu X ping, et al. Depressive neurosis treated by acupuncture for regulating the liver--a report of 176 cases. *J Tradit Chin Med*. 2009;29(2):83-86. doi:10.1016/s0254-6272(09)60037-6

30. Wong YK, Wu JM, Zhou G, et al. Antidepressant Monotherapy and Combination Therapy with Acupuncture in Depressed Patients: A Resting-State Functional Near-Infrared Spectroscopy (fNIRS) Study. *Neurotherapeutics*. 2021;18(4):2651-2663. doi:10.1007/s13311-021-01098-3

31. Zhao FY, Xu Y, Yue LP, et al. Manual acupuncture for patients with major depressive disorder and comorbid insomnia: Evidence from polysomnography and serum biomarkers. *WJAM*. 2020;30(1):5-12. doi:10.1016/j.wjam.2020.02.003

32. Yan L da, Zhou P, Lai MQ, et al. Effect of acupuncture combined with low frequency rTMS on comorbid mild-to-moderate depressive disorder and insomnia: a randomized controlled trial. *Zhongguo Zhen Jiu*. 2023;43(4):374-378. doi:10.13703/j.0255-2930.20220730-k0001

33. Yan L da, Li ZX, Zhang Y, et al. Effect of Shugan Tiaoshen acupuncture combined with western medication on depression-insomnia comorbidity due to COVID-19 quarantine: a multi-central randomized controlled trial. *Zhongguo Zhen Jiu*. 2023;43(3):255-260. doi:10.13703/j.0255-2930.20221030-k0004

34. Gao Y, Tong QY, Ma W, et al. Tiaoqi Jieyu acupuncture for treatment-resistant depression: a randomized controlled trial. *Zhongguo Zhen Jiu*. 2023;43(4):417-421. doi:10.13703/j.0255-2930.20220417-k0003

35. Zhao Y, Zou W, Teng W, Dai XH. Clinical study of Tongdu Tiaoshen acupuncture for the treatment of generalized anxiety disorder. *JCAM*. 2014;30(11):24-26.

36. Chen Y, Fan ZQ. Randomized control study on combination of ear acupoint bloodletting and electroacupuncture for treating mild to moderate generalized anxiety disorder. *J Mod Med Health*. 2013;29(13):1928-1930. doi:10.3969/j.issn.1009-5519.2013.13.004

37. Che LN. Therapeutic effect of acupuncture for the treatment of generalized anxiety disorder patients. *Medical Journal of Chinese People’s Health*. 2015;27(8):86-87. doi:10.3969/j.issn.1672-0369.2015.08.047

38. Wang GM. Acupuncture in the treatment of generalized anxiety disorder: a report of 21 cases. *Henan Traditional Chinese Medicine*. 2007;27(7):55-56. doi:10.3969/j.issn.1003-5028.2007.07.041

39. Zhou XF, Li Y, Zhu H, Chen LL. Impacts of acupuncture at twelve meridians acupoints on brain waves of patients with general anxiety disorder. *Zhongguo Zhen Jiu*. 2013;33(5):395-398.

40. Zheng ZY, Zhu CX. Clinical study on acupuncture using back-shu points for anxiety. *Journal of Liaoning College of TCM*. 2004;6(3):208. doi:10.13194/j.jlunivtcm.2004.03.49.zhengzy.043

41. Zhao RZ, Qin LN, Zhao S. Therapeutic observation of An Shen Jie Lu acupuncture for the treatment of generalized anxiety disorder. *Beijing Journal of Traditional Chinese Medicine*. 2018;37(2):123-125. doi:10.16025/j.1674-1307.2018.02.006

42. Liu EJ, Zhang WL, Wang JB, Zhao FG, Bai YP. Acupuncture combined with cranial electrotherapy stimulation on generalized anxiety disorder: a randomized controlled trial. *Zhongguo Zhen Jiu*. 2020;40(11):1187-1190. doi:10.13703/j.0255-2930.20190917-k0004

43. Liu HJ, Luo WZ, Mei SY, Yuan Q, Jin R. Therapeutic observation of acupuncture for the treatment of generalized anxiety disorder. *Journal of Guangzhou University of Traditional Chinese Medicine*. 2007;24(2):119-122. doi:10.3969/j.issn.1007-3213.2007.02.009

44. Tan TT, Wang D, Huang JK, et al. Modulatory effects of acupuncture on brain networks in mild cognitive impairment patients. *Neural Regen Res*. 2017;12(2):250-258. doi:10.4103/1673-5374.200808

45. Chen YQ, Wu HG, Yin P, Xu J, Huang ET, Xu SF. Tongdu Tiaoshen acupuncture method for mild cognitive impairment: a randomized controlled trial. *Zhongguo Zhen Jiu*. 2019;39(11):1141-1145. doi:10.13703/j.0255-2930.2019.11.001

46. Zhao L, Zhang FW, Zhang H, et al. Mild cognitive impairment disease treated with electroacupuncture: a multi-center randomized controlled trial. *Zhongguo Zhen Jiu*. 2012;32(9):779-784.

47. Zhao L, Zhang FW, Zhang H, et al. Therapy of acusector tongdoutiaosui method on recognitive function of mild recognitive dysfunction patients. *Chinese Journal of Gerontology*. 2010;30(10):1321-1323. doi:10.3969/j.issn.1005-9202.2010.10.001

48. Zhang H, Zhao L, Yang S, et al. Clinical observation on effect of scalp electroacupuncture for mild cognitive impairment. *J Tradit Chin Med*. 2013;33(1):46-50. doi:10.1016/s0254-6272(13)60099-0

49. Liu XP, Liu ZY, Lou JR, et al. Intervention of electrical acupuncture in mild cognitive dysfunction. *Xinjiang Zhongyiyao*. 2010;28(2):25-28.

50. Li YX, Wu MM, Hu SQ, Li S, Wang F. Clinical efficacy and mechanism explore of Yuanluo Tongjing acupuncture method combined with western medicine in the treatment of amnestic mild cognitive impairment. *CJTCMP*. 2023;38(3):1379-1383.

51. Karst M, Reinhard M, Thum P, Wiese B, Rollnik J, Fink M. Needle acupuncture in tension-type headache: a randomized, placebo-controlled study. *Cephalalgia*. 2001;21(6):637-642. doi:10.1046/j.1468-2982.2001.00198.x

52. Wang J, Qin X, Xie W, Wang W. Migraine without aura treated with balance acupuncture therapy:a randomized controlled trial. *Zhongguo Zhen Jiu*. 2017;37(8):805-809. doi:10.13703/j.0255-2930.2017.08.002

53. Zhang YJ, Liu L, Jing XH, Wang LP. Clinical study on the effect of acupuncture on heat pain threshold in migraine without aura. *Chinese Journal of Pain Medicine*. 2019;25(4):276-281. doi:10.3969/j.issn.1006-9852.2019.04.006

54. Wang Y, Xue CC, Helme R, Da Costa C, Zheng Z. Acupuncture for Frequent Migraine: A Randomized, Patient/Assessor Blinded, Controlled Trial with One-Year Follow-Up. *Evid Based Complement Alternat Med*. 2015;2015:920353. doi:10.1155/2015/920353

55. Streng A, Linde K, Hoppe A, et al. Effectiveness and tolerability of acupuncture compared with metoprolol in migraine prophylaxis. *Headache*. 2006;46(10):1492-1502. doi:10.1111/j.1526-4610.2006.00598.x

56. Giannini G, Favoni V, Merli E, et al. A Randomized Clinical Trial on Acupuncture Versus Best Medical Therapy in Episodic Migraine Prophylaxis: The ACUMIGRAN Study. *Front Neurol*. 2020;11:570335. doi:10.3389/fneur.2020.570335

57. Musil F, Pokladnikova J, Pavelek Z, Wang B, Guan X, Valis M. Acupuncture in migraine prophylaxis in Czech patients: an open-label randomized controlled trial. *Neuropsychiatr Dis Treat*. 2018;14:1221-1228. doi:10.2147/NDT.S155119

58. Melchart D, Streng A, Hoppe A, et al. Acupuncture in patients with tension-type headache: randomised controlled trial. *BMJ*. 2005;331(7513):376-382. doi:10.1136/bmj.38512.405440.8F

59. Linde K, Streng A, Jürgens S, et al. Acupuncture for patients with migraine: a randomized controlled trial. *JAMA*. 2005;293(17):2118-2125. doi:10.1001/jama.293.17.2118

60. Sozen A, Yilmaz M, Koyuncuoglu HR, Yurekli VA, Uzar E, Kutluhan S. Effectiveness of acupuncture for the treatment of chronic daily headache: A sham-controlled clinical trial. *Acta Medica Mediterranea*. 2013;29(2):167-172.

61. Cai YW, Pei J, Fu QH, et al. Electroacupuncture at Siguan points for migraine of liver yang hyperactivity: a randomized controlled trial. *Zhongguo Zhen Jiu*. 2022;42(5):498-502. doi:10.13703/j.0255-2930.20210403-0001

62. Ferro EC, Biagini AP, da Silva ÍEF, Silva ML, Silva JRT. The combined effect of acupuncture and Tanacetum parthenium on quality of life in women with headache: randomised study. *Acupunct Med*. 2012;30(4):252-257. doi:10.1136/acupmed-2012-010195

63. Xu S, Yu L, Luo X, et al. Manual acupuncture versus sham acupuncture and usual care for prophylaxis of episodic migraine without aura: multicentre, randomised clinical trial. *BMJ*. 2020;368:m697. doi:10.1136/bmj.m697

64. Zheng H, Gao T, Zheng QH, et al. Acupuncture for Patients With Chronic Tension-Type Headache: A Randomized Controlled Trial. *Neurology*. 2022;99(14):e1560-e1569. doi:10.1212/WNL.0000000000200670

65. Liu L, Lyu TL, Fu MY, et al. Changes in brain connectivity linked to multisensory processing of pain modulation in migraine with acupuncture treatment. *Neuroimage Clin*. 2022;36:103168. doi:10.1016/j.nicl.2022.103168

66. Feng XX, Huang KY, Chen L, Zhou K. Clinical efficacy of the shallow puncture and more-twirling acupuncture method in migraine treatment and its effects on serum 5-HT and β-EP levels. *Technol Health Care*. 2023;31(S1):533-540. doi:10.3233/THC-236047

67. Karst M, Rollnik JD, Fink M, Reinhard M, Piepenbrock S. Pressure pain threshold and needle acupuncture in chronic tension-type headache--a double-blind placebo-controlled study. *Pain*. 2000;88(2):199-203. doi:10.1016/S0304-3959(00)00315-8

68. Allais G, De Lorenzo C, Quirico PE, et al. Non-pharmacological approaches to chronic headaches: transcutaneous electrical nerve stimulation, lasertherapy and acupuncture in transformed migraine treatment. *Neurol Sci*. 2003;24 Suppl 2:S138-142. doi:10.1007/s100720300062

69. Zhao JY, Wang FC. Effects of acupuncturing Sanyinjiao (SP6) on PSQI and PSG in patients with primary insomnia. *CJTCMP*. 2018;33(12):5683-5686.

70. Wang XQ, Qin S, Wu WZ, et al. Effect of electroacupuncture on serum melatonin and dopamine in aged insomnia. *Zhongguo Zhen Jiu*. 2021;41(5):501-504. doi:10.13703/j.0255-2930.20200404-k0001

71. Wang YK, Li T, Ha LJ, et al. Effectiveness and cerebral responses of multi-points acupuncture for primary insomnia: a preliminary randomized clinical trial and fMRI study. *BMC Complement Med Ther*. 2020;20(1):254. doi:10.1186/s12906-020-02969-6

72. Xi HQ, Wu WZ, Liu CY, et al. Effect of acupuncture at Tiaoshen acupoints on hyperarousal state in chronic insomnia. *Zhongguo Zhen Jiu*. 2021;41(3):263-267. doi:10.13703/j.0255-2930.20200303-k0004

73. Xiao BB, Luo XJ, Shen YT. Efficacy observation on refractory insomnia treated with the balance needling therapy. *Zhongguo Zhen Jiu*. 2013;33(2):101-104.

74. Yeung WF, Chung KF, Zhang SP, Yap TG, Law ACK. Electroacupuncture for primary insomnia: a randomized controlled trial. *Sleep*. 2009;32(8):1039-1047. doi:10.1093/sleep/32.8.1039

75. Yin X, Li W, Wu H, et al. Efficacy of Electroacupuncture on Treating Depression-Related Insomnia: A Randomized Controlled Trial. *Nat Sci Sleep*. 2020;12:497-508. doi:10.2147/NSS.S253320

76. Zhang L, Tang Y, Hui R, et al. The effects of active acupuncture and placebo acupuncture on insomnia patients: a randomized controlled trial. *Psychology, Health & Medicine*. 2020;25(10):1201-1215. doi:10.1080/13548506.2020.1738015

77. Zhao YN, Wu WZ, Wang XQ, Xu YQ, Jiang YY, Liu CY. To observe the effect of Tiaoshen acupuncture on hyperarousal in patients with chronic insomnia. *World Science and Technology - Modernization of Traditional Chinese Medicine*. 2021;23(7):2538-2543. doi:10.11842/wst.20200704001

78. Chen XQ, Huang B, Wu X. Acupuncture for sleep disorder in chronic fatigue syndrome: a randomized controlled clinical trial. *Lishizhen Medicine and Materia Medica Research*. 2015;26(7):1669-1672.

79. Luo D, Wu YN, Cai L, et al. Clinical efficacy of liver-soothing and mind-regulating acupuncture for depression related insomnia. *Chinese Journal of Gerontology*. 2017;37(15):3837-3839. doi:10.3969/j.issn.1005-9202.2017.15.088

80. Zhang LX, Zhou SY, Zhen QH, et al. Clinical randomized control study of acupuncture treatment for insomnia with anxiety. *World Chinese Medicine*. 2018;13(7):1570-1574. doi:10.3969/j.issn.1673-7202.2018.07.003

81. Dong B, Chen ZQ, Ma J, Yin P, Li SS, Xu SF. Clinical curative observation of applying acupuncture at Yintang (EX-HN3), Baihui (Du20) and Dazhui (Du14) with periosteal puncture method in the treatment of primary insomnia. *Journal of Sichuan of Traditional Chinese Medicine*. 2018;36(3):176-178.

82. Zhao FY, Zhang ZY, Xia XJ, et al. Memory response to manual acupuncture in chronic insomniacs: evidence from event-related potentials. *Journal of Acupuncture and Tuina Science*. 2018;16(6):382-388. doi:10.1007/s11726-018-1078-3

83. Li Y, Zheng DC. Clinical research of abdominal acupuncture for insomnia. *Liaoning Journal of Traditional Chinese Medicine*. 2010;37(5):914-916.

84. Yin Y, Zhang YP, Yang ZX, Li JY. Penetration needling on complementary acupoints for insomnia. *WJAM*. 2017;27(4):8-12.

85. Chen Q, Chen XJ, Zhou ZY, Chen LF. Efficacy of moxibustion on back-shu points for chronic insomnia: a randomized controlled clinical trial. *Chinese Archives of Traditional Chinese Medicine*. 2013;31(11):2483-2485.

86. Yang ZQ. Combination of behavioral therapy and acupuncture for insomnia caused by incoordination between heart and kidney: clinical observation on 37 cases. *Guiding Journal of Traditional Chinese Medicine and Pharmacy*. 2011;17(2):57-59. doi:10.13862/j.cnki.cn43-1446/r.2011.02.041

87. Zhou DP. Preliminary observation and analysis of clinical efficacy of acupuncture for insomnia. *Contemporary Medicine*. 2016;22(34):24-25. doi:10.3969/j.issn.1009-4393.2016.34.016

88. Liu Y, Feng H, Liu W, et al. Regulation action and nerve electrophysiology mechanism of acupuncture on arousal state in patients of primary insomnia. *Zhongguo Zhen Jiu*. 2017;37(1):19-23. doi:10.13703/j.0255-2930.2017.01.004

89. Pan Y, Luo J, Zhang HL. Study on the effect of acupuncture at Sishencong (EX-HN 1) and Baihui (GV 20) on the serum amino acids neurotransmitters of insomnia patients. *WJAM*. 2017;27(1):23-27. doi:10.1016/S1003-5257(17)30095-8

90. Xu YY, Lin XM. Clinical study on acupuncture of six points in regulating spirit and sleep three-needle points for primary insomnia of liver fire disturbing heart type. *Journal of New Chinese Medicine*. 2020;52(3):151-153. doi:10.13457/j.cnki.jncm.2020.03.045

91. Liu F, Chen TY, Li CY. Clinical efficacy of long-time acupuncture at Sishencong (EX-HN1) during Shen-You time for insomnia. *Xinli Yisheng*. 2016;22(13):140-142.

92. Liu F, Wang L, Wei QP, et al. Clinical curative observation of applying acupuncture at Shen-You time to regulate 196 cases of insomnia. *Journal of Sichuan of Traditional Chinese Medicine*. 2015;33(8):174-176.

93. Tu JH, Chung WC, Yang CY, Tzeng DS. A comparison between acupuncture versus zolpidem in the treatment of primary insomnia. *Asian J Psychiatr*. 2012;5(3):231-235. doi:10.1016/j.ajp.2011.12.003

94. Wu WZ, Zheng SY, Liu CY, et al. Effect of Tongdu Tiaoshen acupuncture on serum GABA and CORT levels in patients with chronic insomnia. *Zhongguo Zhen Jiu*. 2021;41(7):721-724. doi:10.13703/j.0255-2930.20200704-k0001

95. Ji X, Wang Q, Zhu W. Expressions of neurotransmitters in patients of insomnia differentiated as liver stagnation transforming into fire treated with acupuncture. *Zhongguo Zhen Jiu*. 2015;35(6):549-552.

96. Guo J, Wang LP, Wu X. Effect of acupuncture on daytime arousal of patients with primary insomnia. *Beijing Journal of Traditional Chinese Medicine*. 2008;27(7):497-499. doi:10.16025/j.1674-1307.2008.07.009

97. He T, Lai XS, Chen YQ. Acupuncture in the treatment of insomnia complicated with anxiety or depression: a report of 30 cases. *Journal of Anhui TCM College*. 2010;29(1):39-40.

98. Zhao CF, Huang YS. Clinical efficacy of acupuncture for primary insomnia. *Guide of China Medicine*. 2017;15(12):217.

99. Wang YP, Wen X, Feng XL, He TY. Yinyang Ruyin acupuncture on refractory insomnia: a randomized controlled trial. *Zhongguo Zhen Jiu*. 2019;39(11):1155-1159. doi:10.13703/j.0255-2930.2019.11.005

100. Wang J, Wang J, Wang L, Zhang Y. Senile insomnia treated with integrated acupuncture and medication therapy: a randomized controlled trial. *Zhongguo Zhen Jiu*. 2015;35(6):544-548.

101. Zhang X, Han Z, Su T, et al. Combination of acupuncture improves the effect of estazolam on senile insomnia. *Int J Clin Exp Med*. 2019;12(12):13408-13417.

102. Luo WZ, Zhang QZ, Lai XS. Effect of acupuncture treatment of relieving depression and regulating mind on insomnia accompanied with depressive disorders. *Zhongguo Zhen Jiu*. 2010;30(11):899-903.

103. Zhang LX, Hui RT, Tang Y, et al. Effects of acupuncture on insomnia with depression. *CJTCMP*. 2020;35(8):4271-4274.

104. Li X. Effect observation of conical stone needle on foot combined with bian anvil for insomnia. *Zhongguo Zhen Jiu*. 2016;36(2):181-183.

105. Yin X, Gou M, Xu J, et al. Efficacy and safety of acupuncture treatment on primary insomnia: a randomized controlled trial. *Sleep Med*. 2017;37:193-200. doi:10.1016/j.sleep.2017.02.012

106. Feng H, Liu Y, Xu H, Liu YH, Chen GL, Liu WJ. Effect of acupuncture and estazolam on episodic memory and sleep structure in patients with chronic insomnia disorder: a randomized controlled trial. *Zhongguo Zhen Jiu*. 2020;40(7):707-712. doi:10.13703/j.0255-2930.20191208-k0006

107. Wu X, Hu H, Xing J, Zhou M, Xie ZY. Effect of pricking Shenmai (BL 62) and Zhaohai (KI 6) on daytime arousal of patients with chronic insomnia: a randomized controlled trial. *WJAM*. 2014;24(4):1-5+23.

108. Huo YS, Chen ZY, Yin XJ, et al. Tiaoshen acupuncture for primary insomnia: a pilot randomized controlled trial. *Zhongguo Zhen Jiu*. 2023;43(9):1008-1013. doi:10.13703/j.0255-2930.20221120-0001

109. Zhang L, Deng Y, Hui R, et al. The effects of acupuncture on clinical efficacy and steady-state visual evoked potentials in insomnia patients with emotional disorders: A randomized single-blind sham-controlled trial. *Front Neurol*. 2022;13:1053642. doi:10.3389/fneur.2022.1053642

110. Chen Z, Jiang T, Yin X, Li B, Tan Z, Guo J. The increased functional connectivity between the locus coeruleus and supramarginal gyrus in insomnia disorder with acupuncture modulation. *Front Neurosci*. 2023;17:1131916. doi:10.3389/fnins.2023.1131916

**eTable 4. Adverse events summary**

|  | **Local Bleeding** | | **Hematoma** | | **Needling Pain** | | **Dizziness** | | **Others*** | | **Sample** | |  |
| --- | --- | --- | --- | --- | --- | --- | --- | --- | --- | --- | --- | --- | --- |
| **ID** | **A** | **C** | **A** | **C** | **A** | **C** | **A** | **C** | **A** | **C** | **A** | **C** | **Total** |
| 5 | - | - | 1 | 0 | 1 | 0 | - | - | 0 | 1 | 34 | 32 | 66 |
| 13 | - | - | - | - | - | - | - | - | NI | NI | 31 | 29 | 60 |
| 14 | - | - | - | - | - | - | 2 | 0 | 0 | 8 | 32 | 33 | 65 |
| 15 | - | - | 1 | 0 | - | - | - | - | 0 | 4 | 30 | 30 | 60 |
| 16 | - | - | - | - | - | - | 2 | 0 | 0 | NI | 60 | 60 | 120 |
| 19 | - | - | - | - | - | - | 1 | 0 | - | - | 30 | 29 | 59 |
| 20 | - | - | - | - | - | - | 0 | 1 | 2 | 2 | 36 | 34 | 70 |
| 22 | - | - | - | - | - | - | - | - | 1 | 2 | 29 | 25 | 54 |
| 24 | - | - | - | - | - | - | 0 | 1 | 1 | 1 | 23 | 23 | 46 |
| 25 | - | - | 1 | 0 | - | - | - | - | 0 | 1 | 29 | 27 | 56 |
| 28 | - | - | - | - | 1 | 0 | - | - | 0 | 4 | 24 | 24 | 48 |
| 35 | - | - | 1 | 0 | - | - | 1 | 2 | 0 | 6 | 30 | 30 | 60 |
| 37 | - | - | 4 | 0 | - | - | 2 | 4 | 0 | 8 | 40 | 40 | 80 |
| 40 | - | - | - | - | - | - | - | - | 0 | NI | 58 | 50 | 108 |
| 41 | - | - | 1 | 0 | 1 | 0 | 1 | 2 | 0 | 7 | 55 | 59 | 114 |
| 43 | - | - | - | - | - | - | - | - | 1 | 3 | 27 | 26 | 53 |
| 46 | - | - | 5 | 0 | - | - | 3 | 0 | 0 | 9 | 94 | 93 | 187 |
| 47 | - | - | 2 | 0 | - | - | - | - | - | - | 30 | 31 | 61 |
| 50 | - | - | 4 | 0 | - | - | - | - | - | - | 30 | 30 | 60 |
| 55 | - | - | - | - | - | - | - | - | 9 | 34 | 59 | 54 | 113 |
| 56 | - | - | - | - | - | - | - | - | 4 | 2 | 69 | 66 | 135 |
| 57 | - | - | 1 | 0 | - | - | - | - | - | - | 42 | 44 | 86 |
| 58 | - | - | - | - | - | - | - | - | 23 | 11 | 132 | 63 | 195 |
|  | **Local Bleeding** | | **Hematoma** | | **Needling Pain** | | **Dizziness** | | **Symptoms Worsen*** | | **Sample** | |  |
| **ID** | **A** | **C** | **A** | **C** | **A** | **C** | **A** | **C** | **A** | **C** | **A** | **C** | **Total** |
| 59 | - | - | 4 | 2 | - | - | - | - | 16 | 3 | 145 | 81 | 226 |
| 60 | NI | NI | NI | NI | NI | NI | NI | NI | - | - | 34 | 22 | 56 |
| 61 | - | - | 1 | 0 | - | - | - | - | 0 | 2 | 53 | 53 | 106 |
| 63 | - | - | - | - | - | - | - | - | 5 | 0/0 | 58 | 60/29 | 147 |
| 64 | - | - | 1 | 1 | 2 | 0 | - | - | - | - | 110 | 108 | 218 |
| 72 | - | - | 2 | 0 | - | - | - | - | - | - | 29 | 29 | 58 |
| 73 | - | - | - | - | - | - | 1 | 0 | - | - | 20 | 20 | 40 |
| 74 | - | - | 1 | 0 | - | - | - | - | 3 | 3 | 30 | 30 | 60 |
| 75 | - | - | 0 | 1/0 | 3 | 0/0 | 0 | 1/1 | - | - | 30 | 30/30 | 90 |
| 76 | - | - | 2 | 0 | 1 | 2 | 1 | 0 | - | - | 46 | 44 | 90 |
| 79 | - | - | - | - | - | - | - | - | 1 | 0 | 36 | 34 | 70 |
| 80 | - | - | 3 | 0 | - | - | - | - | - | - | 33 | 34 | 67 |
| 82 | - | - | 1 | 0 | - | - | - | - | - | - | 30 | 30 | 60 |
| 83 | - | - | 2 | 1/3 | - | - | - | - | - | - | 30 | 30/30 | 90 |
| 85 | 3 | 0 | - | - | - | - | 0 | 3 | 0 | 8 | 59 | 57 | 116 |
| 91 | - | - | - | - | - | - | 1 | 0 | - | - | 32 | 31 | 63 |
| 92 | - | - | - | - | - | - | 2 | 0 | - | - | 96 | 95 | 191 |
| 100 | - | - | - | - | - | - | - | - | 0 | NI | 33 | 30 | 63 |
| 102 | - | - | - | - | - | - | - | - | 4 | NI | 33 | 32 | 65 |
| 103 | 3 | 0 | - | - | 1 | 2 | - | - | - | - | 30 | 30 | 60 |
| 105 | - | - | 1 | 0 | - | - | - | - | 1 | 0 | 36 | 36 | 72 |
| 106 | - | - | 1 | 0/1 | - | - | 0 | 0/1 | 0 | 2/0 | 45 | 45/45 | 135 |

Abbreviations: A, acupuncture group; C, control group; NI, no information.

*Including headache, fatigue, insomnia, etc. / intolerant to intervention / non-specific reason.

**eTable 5. PRISMA 2020 Checklist**

| **Section and Topic** | **Item #** | **Checklist item** | **Location** |
| --- | --- | --- | --- |
| **TITLE** | | |  |
| Title | 1 | Identify the report as a systematic review. | P.1 |
| **ABSTRACT** | | |  |
| Abstract | 2 | See the PRISMA 2020 for Abstracts checklist. | P.4 |
| **INTRODUCTION** | | |  |
| Rationale | 3 | Describe the rationale for the review in the context of existing knowledge. | P.6 |
| Objectives | 4 | Provide an explicit statement of the objective(s) or question(s) the review addresses. | P.7 |
| **METHODS** | | |  |
| Eligibility criteria | 5 | Specify the inclusion and exclusion criteria for the review and how studies were grouped for the syntheses. | P.7-8 |
| Information sources | 6 | Specify all databases, registers, websites, organisations, reference lists and other sources searched or consulted to identify studies. Specify the date when each source was last searched or consulted. | P.8 |
| Search strategy | 7 | Present the full search strategies for all databases, registers and websites, including any filters and limits used. | eTable 1 |
| Selection process | 8 | Specify the methods used to decide whether a study met the inclusion criteria of the review, including how many reviewers screened each record and each report retrieved, whether they worked independently, and if applicable, details of automation tools used in the process. | P.8 |
| Data collection process | 9 | Specify the methods used to collect data from reports, including how many reviewers collected data from each report, whether they worked independently, any processes for obtaining or confirming data from study investigators, and if applicable, details of automation tools used in the process. | P.8-9 |
| Data items | 10a | List and define all outcomes for which data were sought. Specify whether all results that were compatible with each outcome domain in each study were sought (e.g. for all measures, time points, analyses), and if not, the methods used to decide which results to collect. | P.8 |
|  | 10b | List and define all other variables for which data were sought (e.g. participant and intervention characteristics, funding sources). Describe any assumptions made about any missing or unclear information. | P.8 |
| Study risk of bias assessment | 11 | Specify the methods used to assess risk of bias in the included studies, including details of the tool(s) used, how many reviewers assessed each study and whether they worked independently, and if applicable, details of automation tools used in the process. | P.8 |
| Effect measures | 12 | Specify for each outcome the effect measure(s) (e.g. risk ratio, mean difference) used in the synthesis or presentation of results. | P.8 |
| Synthesis methods | 13a | Describe the processes used to decide which studies were eligible for each synthesis (e.g. tabulating the study intervention characteristics and comparing against the planned groups for each synthesis (item #5)). | P.8 |
|  | 13b | Describe any methods required to prepare the data for presentation or synthesis, such as handling of missing summary statistics, or data conversions. | NA |
|  | 13c | Describe any methods used to tabulate or visually display results of individual studies and syntheses. | P.8 |
|  | 13d | Describe any methods used to synthesize results and provide a rationale for the choice(s). If meta-analysis was performed, describe the model(s), method(s) to identify the presence and extent of statistical heterogeneity, and software package(s) used. | P.8 |
|  | 13e | Describe any methods used to explore possible causes of heterogeneity among study results (e.g. subgroup analysis, meta-regression). | P.9 |
|  | 13f | Describe any sensitivity analyses conducted to assess robustness of the synthesized results. | P.9 |
| Reporting bias assessment | 14 | Describe any methods used to assess risk of bias due to missing results in a synthesis (arising from reporting biases). | P.9 |
| Certainty assessment | 15 | Describe any methods used to assess certainty (or confidence) in the body of evidence for an outcome. | NA |
| **RESULTS** | | |  |
| Study selection | 16a | Describe the results of the search and selection process, from the number of records identified in the search to the number of studies included in the review, ideally using a flow diagram. | P.9 |
|  | 16b | Cite studies that might appear to meet the inclusion criteria, but which were excluded, and explain why they were excluded. | Figure 1 |
| Study characteristics | 17 | Cite each included study and present its characteristics. | eTable 3 |
| Risk of bias in studies | 18 | Present assessments of risk of bias for each included study. | eFigure 1 |
| Results of individual studies | 19 | For all outcomes, present, for each study: (a) summary statistics for each group (where appropriate) and (b) an effect estimate and its precision (e.g. confidence/credible interval), ideally using structured tables or plots. | eFigure 2 |
| Results of syntheses | 20a | For each synthesis, briefly summarise the characteristics and risk of bias among contributing studies. | P.10 |
|  | 20b | Present results of all statistical syntheses conducted. If meta-analysis was done, present for each the summary estimate and its precision (e.g. confidence/credible interval) and measures of statistical heterogeneity. If comparing groups, describe the direction of the effect. | P.10-17 |
|  | 20c | Present results of all investigations of possible causes of heterogeneity among study results. | P.10-17 |
|  | 20d | Present results of all sensitivity analyses conducted to assess the robustness of the synthesized results. | P.12,16 |
| Reporting biases | 21 | Present assessments of risk of bias due to missing results (arising from reporting biases) for each synthesis assessed. | P.10 |
| Certainty of evidence | 22 | Present assessments of certainty (or confidence) in the body of evidence for each outcome assessed. | P.18 |
| **DISCUSSION** | | |  |
| Discussion | 23a | Provide a general interpretation of the results in the context of other evidence. | P.18-20 |
|  | 23b | Discuss any limitations of the evidence included in the review. | P.21 |
|  | 23c | Discuss any limitations of the review processes used. | P.21 |
|  | 23d | Discuss implications of the results for practice, policy, and future research. | P.20-21 |
| **OTHER INFORMATION** | | |  |
| Registration and protocol | 24a | Provide registration information for the review, including register name and registration number, or state that the review was not registered. | P.5 |
|  | 24b | Indicate where the review protocol can be accessed, or state that a protocol was not prepared. | NA |
|  | 24c | Describe and explain any amendments to information provided at registration or in the protocol. | NA |
| Support | 25 | Describe sources of financial or non-financial support for the review, and the role of the funders or sponsors in the review. | P.22 |
| Competing interests | 26 | Declare any competing interests of review authors. | P.22 |
| Availability of data, code and other materials | 27 | Report which of the following are publicly available and where they can be found: template data collection forms; data extracted from included studies; data used for all analyses; analytic code; any other materials used in the review. | P.22 |

**eFigure 1. Risk of bias assessment of included studies**

| **Unique ID** | **Study ID** | **D1** | **D2** | **D3** | **D4** | **D5** | **Overall** |  |  |  |
| --- | --- | --- | --- | --- | --- | --- | --- | --- | --- | --- |
| 1 | Wang, et al. | 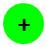 | 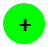 | 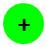 | 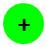 | 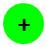 | 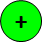 |  | 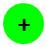 | Low risk |
| 2 | Chen, et al. | 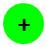 | 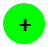 | 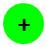 | 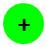 | 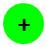 | 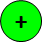 |  | 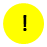 | Some concerns |
| 3 | Zheng, et al. | 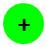 | 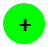 | 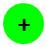 | 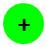 | 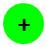 | 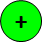 |  | 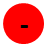 | High risk |
| 4 | An, et al. | 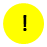 | 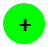 | 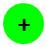 | 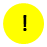 | 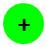 | 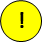 |  |  |  |
| 5 | Wu, et al. | 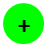 | 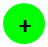 | 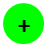 | 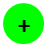 | 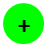 | 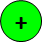 |  | D1 | Randomisation process |
| 6 | Ma, et al. | 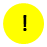 | 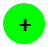 | 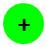 | 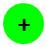 | 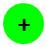 | 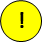 |  | D2 | Deviations from the intended interventions |
| 7 | Tian, et al. | 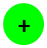 | 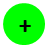 | 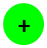 | 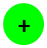 | 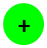 | 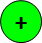 |  | D3 | Missing outcome data |
| 8 | Song, et al. | 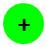 | 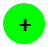 | 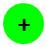 | 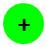 | 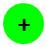 | 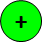 |  | D4 | Measurement of the outcome |
| 9 | Zhu, et al. | 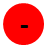 | 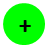 | 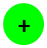 | 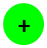 | 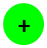 | 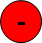 |  | D5 | Selection of the reported result |
| 10 | Jiang, et al. | 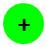 | 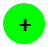 | 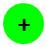 | 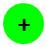 | 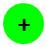 | 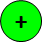 |  |  |  |
| 11 | Li, et al. | 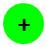 | 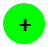 | 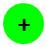 | 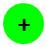 | 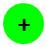 | 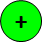 |  |  |  |
| 12 | Li, et al. | 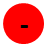 | 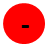 | 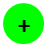 | 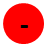 | 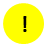 | 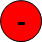 |  |  |  |
| 13 | Ma, et al. | 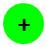 | 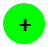 | 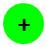 | 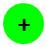 | 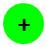 | 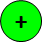 |  |  |  |
| 14 | Wei, et al. | 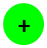 | 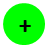 | 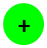 | 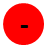 | 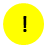 | 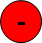 |  |  |  |
| 15 | Yang, et al. | 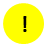 | 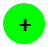 | 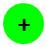 | 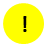 | 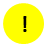 | 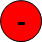 |  |  |  |
| 16 | Xie, et al. | 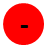 | 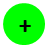 | 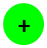 | 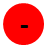 | 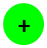 | 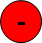 |  |  |  |
| 17 | Yan, et al. | 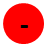 |  |  |  |  |  |  |  |  |
| 18 | Zhang, et al. |  |  |  |  |  |  |  |  |  |
| 19 | Zhu, et al. |  |  |  |  |  |  |  |  |  |
| 20 | 1600 |  |  |  |  |  |  |  |  |  |
| 21 | Luo, et al. |  |  |  |  |  |  |  |  |  |
| 22 | Du, et al. |  |  |  |  |  |  |  |  |  |
| 23 | Yi, et al. |  |  |  |  |  |  |  |  |  |
| 24 | Duan, et al. |  |  |  |  |  |  |  |  |  |
| 25 | Liu, et al. |  |  |  |  |  |  |  |  |  |
| 26 | Ma, et al. |  |  |  |  |  |  |  |  |  |
| 27 | Yi, et al. |  |  |  |  |  |  |  |  |  |
| 28 | Wang, et al. |  |  |  |  |  |  |  |  |  |
| 29 | Fu, et al. |  |  |  |  |  |  |  |  |  |
| 30 | Wong, et al. |  |  |  |  |  |  |  |  |  |
| 31 | Zhao, et al. |  |  |  |  |  |  |  |  |  |
| 32 | Yan, et al. |  |  |  |  |  |  |  |  |  |
| 33 | Yan, et al. |  |  |  |  |  |  |  |  |  |
| 34 | Gao, et al. |  |  |  |  |  |  |  |  |  |
| 35 | Zhao, et al. |  |  |  |  |  |  |  |  |  |
| 36 | Chen, et al. |  |  |  |  |  |  |  |  |  |
| 37 | Che, et al. |  |  |  |  |  |  |  |  |  |
| 38 | Wang, et al. |  |  |  |  |  |  |  |  |  |
| 39 | Zhou, et al. |  |  |  |  |  |  |  |  |  |
| 40 | Zheng, et al. |  |  |  |  |  |  |  |  |  |
| 41 | Zhao, et al. |  |  |  |  |  |  |  |  |  |
| 42 | Liu, et al. |  |  |  |  |  |  |  |  |  |
| 43 | Liu, et al. |  |  |  |  |  |  |  |  |  |
| 44 | Tan, et al. |  |  |  |  |  |  |  |  |  |
| 45 | Chen, et al. |  |  |  |  |  |  |  |  |  |
| 46 | Zhao, et al. |  |  |  |  |  |  |  |  |  |
| 47 | Zhao, et al. |  |  |  |  |  |  |  |  |  |
| 48 | Zhang, et al. |  |  |  |  |  |  |  |  |  |
| 49 | Liu, et al. |  |  |  |  |  |  |  |  |  |
| 50 | Li, et al. |  |  |  |  |  |  |  |  |  |
| 51 | Karst, et al. |  |  |  |  |  |  |  |  |  |
| 52 | Wang, et al. |  |  |  |  |  |  |  |  |  |
| 53 | Zhang, et al. |  |  |  |  |  |  |  |  |  |
| 54 | Wang, et al. |  |  |  |  |  |  |  |  |  |
| 55 | Streng, et al. |  |  |  |  |  |  |  |  |  |
| 56 | Giannini, et al. |  |  |  |  |  |  |  |  |  |
| 57 | Musil, et al. |  |  |  |  |  |  |  |  |  |
| 58 | Melchart, et al. |  |  |  |  |  |  |  |  |  |
| 59 | Linde, et al. |  |  |  |  |  |  |  |  |  |
| 60 | Sozen, et al. |  |  |  |  |  |  |  |  |  |
| 61 | Cai, et al. |  |  |  |  |  |  |  |  |  |
| 62 | Ferro, et al. |  |  |  |  |  |  |  |  |  |
| 63 | Xu, et al. |  |  |  |  |  |  |  |  |  |
| 64 | Zheng. et al. |  |  |  |  |  |  |  |  |  |
| 65 | Liu, et al. |  |  |  |  |  |  |  |  |  |
| 66 | Feng, et al. |  |  |  |  |  |  |  |  |  |
| 67 | Karst, et al. |  |  |  |  |  |  |  |  |  |
| 68 | Allais, et al. |  |  |  |  |  |  |  |  |  |
| 69 | Zhao, et al. |  |  |  |  |  |  |  |  |  |
| 70 | Wang, et al. |  |  |  |  |  |  |  |  |  |
| 71 | Wang, et al. |  |  |  |  |  |  |  |  |  |
| 72 | Xi, et al. |  |  |  |  |  |  |  |  |  |
| 73 | Xiao, et al. |  |  |  |  |  |  |  |  |  |
| 74 | Yeung, et al. |  |  |  |  |  |  |  |  |  |
| 75 | Yin, et al. |  |  |  |  |  |  |  |  |  |
| 76 | Zhang, et al. |  |  |  |  |  |  |  |  |  |
| 77 | Zhao, et al. |  |  |  |  |  |  |  |  |  |
| 78 | Chen, et al. |  |  |  |  |  |  |  |  |  |
| 79 | Luo, et al. |  |  |  |  |  |  |  |  |  |
| 80 | Zhang, et al. |  |  |  |  |  |  |  |  |  |
| 81 | Dong, et al. |  |  |  |  |  |  |  |  |  |
| 82 | Zhao, et al. |  |  |  |  |  |  |  |  |  |
| 83 | Li, et al. |  |  |  |  |  |  |  |  |  |
| 84 | Yin, et al. |  |  |  |  |  |  |  |  |  |
| 85 | Chen, et al. |  |  |  |  |  |  |  |  |  |
| 86 | Yang, et al. |  |  |  |  |  |  |  |  |  |
| 87 | Zhou, et al. |  |  |  |  |  |  |  |  |  |
| 88 | Liu, et al. |  |  |  |  |  |  |  |  |  |
| 89 | Pan, et al. |  |  |  |  |  |  |  |  |  |
| 90 | Xu, et al. |  |  |  |  |  |  |  |  |  |
| 91 | Liu, et al. |  |  |  |  |  |  |  |  |  |
| 92 | Liu, et al. |  |  |  |  |  |  |  |  |  |
| 93 | Tu, et al. |  |  |  |  |  |  |  |  |  |
| 94 | Wu, et al. |  |  |  |  |  |  |  |  |  |
| 95 | Ji, et al. |  |  |  |  |  |  |  |  |  |
| 96 | Guo, et al. |  |  |  |  |  |  |  |  |  |
| 97 | He, et al. |  |  |  |  |  |  |  |  |  |
| 98 | Zhao, et al. |  |  |  |  |  |  |  |  |  |
| 99 | Wang, et al. |  |  |  |  |  |  |  |  |  |
| 100 | Wang, et al. |  |  |  |  |  |  |  |  |  |
| 101 | Zhang, et al. |  |  |  |  |  |  |  |  |  |
| 102 | Luo, et al. |  |  |  |  |  |  |  |  |  |
| 103 | Zhang, et al. |  |  |  |  |  |  |  |  |  |
| 104 | Li, et al. |  |  |  |  |  |  |  |  |  |
| 105 | Yin, et al. |  |  |  |  |  |  |  |  |  |
| 106 | Feng, et al. |  |  |  |  |  |  |  |  |  |
| 107 | Wu, et al. |  |  |  |  |  |  |  |  |  |
| 108 | Huo, et al |  |  |  |  |  |  |  |  |  |
| 109 | Zhang, et al. |  |  |  |  |  |  |  |  |  |
| 110 | Chen, et al. |  |  |  |  |  |  |  |  |  |

**eFigure 2. Subgroup analysis**

**1. Depression**

**Outcome: Hamilton Depression Scale (HAMD)**

**AC (Acupuncture) vs Medication (by Treatment Duration)**

**AC vs Medication (by Type of AC)**

**AC vs Medication (by Type of Medication)**

**2. Insomnia**

**Outcome: Hamilton Depression Scale (PSQI)**

**AC vs Medication (by Type of AC)**

**AC vs Medication (by Type of Medication)**

**AC vs Sham-AC (by Treatment Duration)**

**AC vs Sham-AC (by Type of AC)**

**eFigure 3. Sensitivity analysis**

**1. Depression**

**Outcome: Hamilton Depression Scale (HAMD)**

**AC (Acupuncture) vs Medication**

**2. Insomnia**

**Outcome: Hamilton Depression Scale (PSQI)**

**AC vs Medication**

**AC vs Sham-AC**

**eFigure 4. Funnel plots**

**1. Depression**

**Outcome: Hamilton Depression Scale (HAMD)**

**Acupuncture vs Medication**

**2. Insomnia**

**Outcome: Hamilton Depression Scale (PSQI)**

**Acupuncture vs Medication**

**Acupuncture vs Sham Acupuncture**
